# A better index for analysis of co-occurrence and similarity

**DOI:** 10.1126/sciadv.abj9204

**Published:** 2022-01-26

**Authors:** Kumar P. Mainali, Eric Slud, Michael C. Singer, William F. Fagan

**Affiliations:** 1 National Socio-Environmental Synthesis Center (SESYNC), University of Maryland, 1 Park Pl Suite 300, Annapolis, MD 21401, USA.; 2 Conservation Innovation Center, Chesapeake Conservancy, 716 Giddings Ave Suite 42, Annapolis, MD 21403, USA.; 3 Department of Biology, University of Maryland, 1210 Biology-Psychology Building, College Park, MD 20742, USA.; 4 Department of Mathematics, University of Maryland, College Park, MD 20742, USA.; 5 Center for Statistical Research and Methodology, U.S. Census Bureau, 4600 Silver Hill Road, Washington, DC 20233, USA.; 6 Station CNRS d’Écologie Théorique et Expérimentale, 09200 Moulis, France.

## Abstract

Scientists often need to know whether pairs of entities tend to occur together or independently. Standard approaches to this issue use co-occurrence indices such as Jaccard, Sørensen-Dice, and Simpson. We show that these indices are sensitive to the prevalences of the entities they describe and that this invalidates their interpretability. We propose an index, α, that is insensitive to prevalences. Published datasets reanalyzed with both α and Jaccard’s index (*J*) yield profoundly different biological inferences. For example, a published analysis using *J* contradicted predictions of the island biogeography theory finding that community stability increased with increasing physical isolation. Reanalysis of the same dataset with the estimator $\hat{\alpha }$ reversed that result and supported theoretical predictions. We found similarly marked effects in reanalyses of antibiotic cross-resistance and human disease biomarkers. Our index α is not merely an improvement; its use changes data interpretation in fundamental ways.

## Teaser

Better similarity analyses fundamentally change the interpretation of results in diverse biological datasets.

## INTRODUCTION

The probabilistic analysis of co-occurrence dates back to at least the 18th century (*1*). Subsequently, statistical presentation and analysis of contingency tables began in the 19th century (*2*, *3*) and formal statistics in the 20th century (*4*–*6*). Nowadays, biologists regularly use metrics of association or co-occurrence to quantify similarities and differences among sets of observations—be they communities, diseases, or genes (*7*–*10*). These metrics are important in diverse biological disciplines, including biogeography (*11*, *12*), biodiversity (*7*, *13*), ecology (*14*), epidemiology (*15*), evolution (*16*), and neuroscience (*17*). Researchers use co-occurrence analyses to quantify species loss and gain across communities (*7*, *8*), to investigate patterns of antibiotic cross-resistance (*9*), and to identify mechanistic similarities among diseases (*10*).

Three of the most popular co-occurrence or association metrics are due to Jaccard, Sørensen-Dice, and Simpson (*18*). For all three metrics, researchers interpret values near zero as representing highly negative associations and those near one as highly positive associations. Across pairs, researchers interpret identical values of a given index as indicating the same level of association. Here, we show how very wrong this interpretation can be. Despite their long history and broad usage, including routine appearance in high-profile papers (section S1), all three metrics, and numerous related measures (*18*), suffer from a major statistical flaw that not only biases their interpretations but also completely invalidates them. As we will show, the source of this problem is the extent to which the numerical values of these metrics are sensitive to the prevalences of their component elements.

**Fig. 1.**
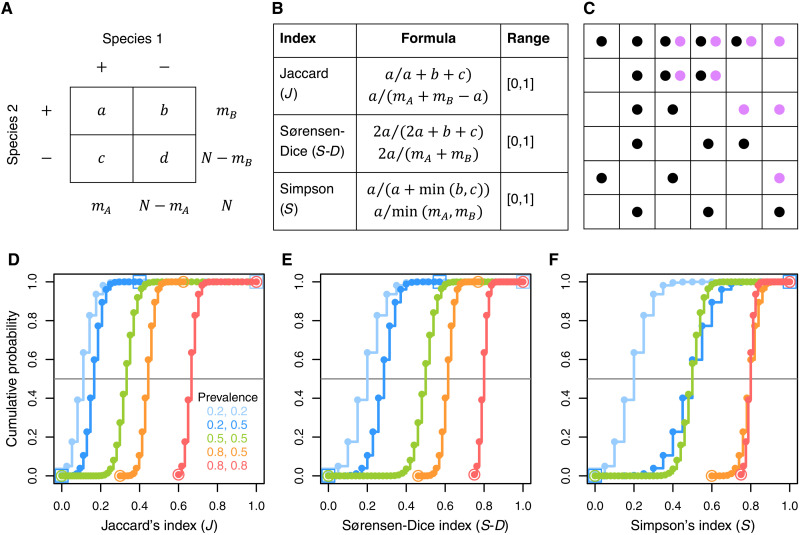
Common measures of association in co-occurrence data and their dependence on prevalence. For the association between species 1 and species 2, the prevalence and co-occurrence (**A**) are used to compute metrics of association (**B**). The probability of an observed co-occurrence of mutually noninfluencing species can be determined when occupancy of the sites by species 1 (black balls) and species 2 (purple balls) is random, with fixed prevalences (**C**). Within a classic framework of balls (species) placed independently in boxes (sites) with fixed marginals (species prevalence), the probability distribution of species co-occurrences is uniquely determined as hypergeometric (see Materials and Methods). Cumulative distribution functions for similarity metrics (five prevalence scenarios with two species independently distributed across 100 sites) demonstrate that the same metric value corresponds to different probabilities based on prevalence (**D** to **F**); empty symbol in a color represents the theoretical maximum and minimum of the similarity metrics for the respective prevalence scenario.

To illustrate this, we construct an ecological example involving a grid of *N* sites, of which *m_A_* contain species 1 and *m_B_* contain species 2. Species 1’s prevalence is *m_A_*/*N*. Suppose further that *a* sites contain both species, *d* sites contain neither, *b* sites contain only species 2, and *c* sites contain only species 1 (Fig. 1A). Calculations of each of the three indices (Fig. 1B) vary with prevalence (Fig. 1, D to F). Because the ranges and midpoints of the indices depend strongly on prevalence, they yield misleading inferences about species association. We explain below how the same metric value can indicate strong negative association in one scenario of prevalence (e.g., the Jaccard’s index *J* = 0.6 in the red curve) and strong positive association in another scenario (*J* = 0.6 in the orange curve).

### Roadmap to the reliable analysis of co-occurrence data

The analysis of co-occurrence data is over a century old, resulting in ~80 metrics of association (*19*). Within biogeography and ecology, many problems associated with these metrics have emerged in the past several decades, and many solutions to those problems have been proposed. Below, we summarize in five extended points the fundamental nature of the problem stemming from the structure of the null distribution, the main developments in co-occurrence analysis, and their strengths and shortcomings.

1) Identifying a null distribution. We adopt four classic assumptions for our null hypothesis: first, that available sites are exchangeable in the sense that each one is equally likely to be selected by species A and B; second, that, in the selection of sites, each species acts independently of the other; third, that both the total number of sites (*N*) and the prevalences of the two species (*m_A_* and *m_B_*) are fixed; and fourth, that the occurrences are observed without error. Under these assumptions, as we will show, there is only one mathematically correct distribution for the number of co-occurring sites when the prevalences *m_A_* and *m_B_* of the two species and the number of sites are fixed. This distribution is hypergeometric (*N*, *m_A_*, *m_B_*).

Within ecological literature, only Veech (*20*) and Griffith *et al.* (*21*) recognized the correct mathematical form of the null distribution. Veech (*20*) developed a probabilistic model of co-occurrence in this null situation, and Griffith *et al.* (*21*) correctly identified that the combinations in Veech’s solution simplify to a hypergeometric (*N*, *m_A_*, *m_B_*) distribution.

2) Simulation. Several studies have simulated the null distribution. For example, Keil (*19*) correctly simulated null configurations according to the assumptions in #1. So, his calculations of null hypothesis probabilities (and means and variances) are, in principle, correct, but they may still be numerically inaccurate depending on how many randomization replications are used and how extreme is the desired tail probability for the observed numbers of co-occurrences. Simulation via randomization of the data is also often used to approximate the null distribution, and considerable debate exists concerning which aspect(s) of data should be randomized and which should be held fixed in building the null models (*22*, *23*). However, under the four assumptions of #1, there is only one correct answer for the null distribution.

3) Standardization of counts and indices. In practice, co-occurrence counts depend strongly on the prevalences *m_A_* and *m_B_*. In principle, standardization of counts and the indices (*19*, *24*, *25*) can mitigate this dependence, and prior studies have shown that dependence can indeed be reduced (*19*, *23*). However, in practice, standardizations fail to completely remove confounding effects of variation in both prevalences and species richness (*22*).

Specifically, reliance on standardized counts in settings where the hypergeometric is not well approximated by a normal distribution does not effectively reveal how extreme the co-occurrence value is with respect to the null hypothesis tail probability. This is because standardizing observations using a non-normal, skewed reference distribution gives an inadequate summary of how extreme observations are in the reference distribution tails. Furthermore, measures of non-nullity are especially inaccurate when species-pair data exhibit strong (positive or negative) associations. Collectively, these deficiencies (listed under #2 and #3) point to the need for analyses of co-occurrence that are rooted in a precise statistical distribution, not in simulation-based approaches.

4) Lack of *P* value standardization. Using *P* values as a standard summary statistic would remove the dependence on prevalence and would not suffer from the objection in #3, but researchers have apparently not found these *P* values to be an appealing or interpretable summary of species-pair association.

5) Mechanistic associations. Neither of the standardizations in #3 and #4 adequately summarizes the co-occurrences in non-null situations across different prevalence combinations, when there is a definite mechanism of association, meaning that there are specific different probabilities for species B to occupy a site according to whether species A occupies that site. When a single study contains many simultaneous species-pair or other entities’ comparisons, a researcher wants to do much more than a hypothesis test using *P* values for each comparison. An effective quantitative grouping of pairs with similar degrees of association and quantifying degrees of non-null association is not something that can be achieved with null distribution *P* values or null distribution standardizations.

Collectively, half a century of development in analyses of co-occurrence has been marred by failures to identify the null model, attempts at standardization that are misleading when sample sizes are small and distributions are discrete and skewed, and the lack of development of a statistic that reliably identifies departures from expected co-occurrence patterns. Progress on the issue has been piecemeal. Some researchers developed an approach for constructing a null model with simulation (*19*) and others found the mathematical form of the null model (*20*, *21*). MacKenzie *et al.* [(*26*), chapter 8] introduced an interpretable parameter governing the mechanism of co-occurrence but did not make the connection to null and alternative distributions for indices similar to those of Jaccard and Sørensen–Dice. However, none of these advances offers a complete solution because measuring non-nullity using null distribution descriptive statistics is not helpful in quantifying departures from nullity, although it is useful in hypothesis testing. Here, we resolve all the aforementioned challenges and present a meaningful and interpretable parameter of association in binary co-occurrence data, one that emerges from solid statistical theory and has a complete mathematical formulation for its probability distribution.

### Null model

If the two species independently assort their respective site counts *m_A_* and *m_B_* equiprobably among all available sites in the grid (Fig. 1C), then the hypergeometric distribution

$$\begin{array}{c} P(X=k)=\left(\frac{m_{A}}{k})(\frac{N-m_{A}}{m_{B}-k}\right)/\left(\frac{N}{m_{B}}\right),\\ \text{max}(m_{A}+m_{B}-N,0)\leq k\leq \text{min}(m_{A},m_{B}) \end{array}$$ (1)

is the only possible reference distribution for co-occurrences. Note that *k* here plays the same role as *a* in Fig. 1A. This distribution was known to Huygens as early as 1657 in a gambling context (*1*) and was proposed as the basis for Fisher’s (*5*) famous “exact test” for independence of row and column categories in a two by two contingency table, following Pearson’s (*4*) earlier use of the chi-square large-sample test of the same hypothesis. Recent work introduced the hypergeometric as a null distribution in ecology (*20*, *21*).

On the basis of two by two table entries in which two species are mutually noninfluencing and have occurrence counts *m_A_* and *m_B_* among *N* sites (Fig. 1A), consider a metric of association *g*(*a*, *b*, *c*, *d*) (e.g., Fig. 1B). The cumulative probability [cumulative distribution function (CDF)] of this metric being ≤*t* is calculated as the sum of probabilities (Eq. 1) for which

$$g(k,m_{B}-k,m_{A}-k,N-m_{A}-m_{B}+k)\leq t$$

Figure 1 (D to F) plots this CDF for three common choices of *g* (Jaccard, Sørensen-Dice, and Simpson), for *N* = 100 and five pairs of prevalences. The horizontal black line denotes a cumulative probability of 0.5, indicating no relationship between species. Under the null distribution for independently acting species, the CDF curves should cross this line at the same location on the *x* axis regardless of prevalence. However, Fig. 1 (D to F) demonstrates that these crossing points vary markedly as a function of prevalence for all three indices. This dependence on prevalence is the crux of the problem with these long-standing and heavily used metrics.

This analysis demonstrates the need for a statistic of association that is insensitive to underlying prevalences. Metric values near the median of the null occurrence distribution should correspond uniformly to lack of association between species, whereas very large and small metric values should always correspond to positive and negative associations between species, respectively.

### The affinity model

We develop a statistical parameter that can be estimated from occurrence data to model a species’ tendency to be present or absent in a habitat patch already occupied by a different species. Consider the scenario from Fig. 1A and assume that *m_A_* sites occupied by species 1 (out of *N* total) are already fixed. Suppose that species 2 will occupy sites already occupied by species 1 with a higher probability compared to sites not occupied by species 1. Suppose that, at each site, independently of all others, species 2 occupies a site with probability *p*_1_ if species 1 is present but with probability *p*_2_ if species 1 is absent. Then, we can quantify the degree of difference of the two probabilities through the log odds ratio

$$\alpha =\text{log}(\frac{p_{1}}{1-p_{1}}/\frac{p_{2}}{1-p_{2}})$$ (2)

where the statistical parameter α quantifies the affinity of the two species for each other; in other words, their tendency to co-occur. Next, to overcome the objection that in co-occurrence studies the number *m_B_* of sites occupied by species 2 should often be regarded as fixed at *m_B_* rather than random, we consider the probabilities *p*_1_ and *p*_2_ as being conditional on a total of *m_B_* sites occupied by species 2. The log odds ratio is the basis for discussions of factorial interactions in multiway contingency tables introduced through log-linear models by Bishop *et al.* (*27*). Its relation to cell probabilities under alternative factor levels in contingency tables is a staple of statistical pedagogy regarding categorical data analysis (*28*) and was already well understood by Fisher (*5*, *6*) and Cornfield (*15*) in two by two tables.

In Materials and Methods, we show that this conditional distribution depends only on *m_A_*, *m_B_*, *N*, and α, but not on *p*_1_ or *p*_2_, and that the same conditional co-occurrence distribution arises if we switch the species’ roles. The resulting distribution has the same range of values as the hypergeometric and agrees with it precisely when α = 0, having the more general form

$$P(X=k)=(\frac{m_{A}}{k})(\frac{N-m_{A}}{m_{B}-k})\;e^{\alpha k}/\sum_{j=0}^{m_{B}}\;(\frac{m_{A}}{j})(\frac{N-m_{A}}{m_{B}-j})e^{\alpha j}$$ (3)

for max(*m_A_* + *m_B_* − *N*,0) ≤ *k* ≤ min (*m_A_*, *m_B_*). This is the extended hypergeometric (*29*) or noncentral hypergeometric (*6*) distribution. Cornfield (*15*) used this for retrospective case-control studies analyzing the odds of disease development.

When two species co-occur more often than the mean of the null hypergeometric distribution, we expect the log odds ratio α to be positive. Similarly, a co-occurrence number less than the mean suggests a negative α. We treat the actual log odds ratio α as an unknown statistical parameter characterizing a species pair, and we estimate it with fixed occurrence counts *m_A_* and *m_B_*, as well as *N* total sites, by maximizing the likelihood (Eq. 3 with *X* = *a* substituted for *k*) as a function of α. The maximum likelihood (ML) estimate of α is $\hat{\alpha }$, and we term it an affinity metric of co-occurrence (see Materials and Methods for derivation).

**Fig. 2.**
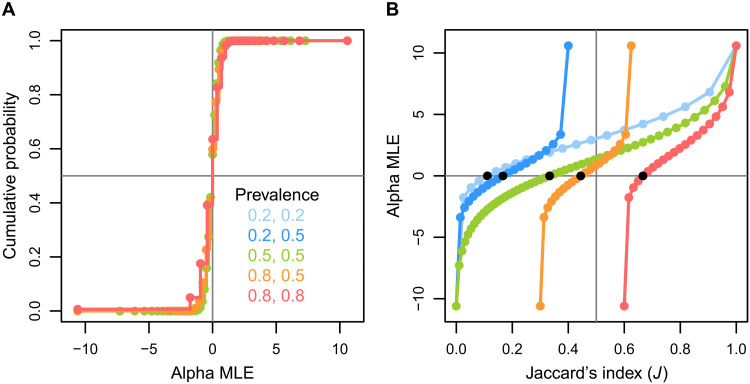
Alpha (α), the statistical parameter that we developed for estimating association of co-occurring entities, and the lack of consistent relationship of Jaccard’s index (*J*), a common traditional index with α. Cumulative distribution function of $\hat{\alpha }$ [α MLE; the maximum likelihood estimates (MLE) of log odds ratio] showing its insensitivity to prevalence (**A**), and the unreliable relationship between $\hat{\alpha }$ and *J*. In (**B**), black-filled circles represent null expectations, indicating multiple mappings of *J* onto $\hat{\alpha }$. Prevalence out of 100 sites.

Contrary to Fig. 1 (D to F), the CDF of $\hat{\alpha }$ is always centered at 0 irrespective of prevalence (Fig. 2A). In summary, $\hat{\alpha }$ has a defined probability mass function, which is insensitive to prevalences under the null model where α = 0, and estimates the parameter α quantifying the preferential selection of occupied sites. Mapping *J* against $\hat{\alpha }$ (Fig. 2B) shows that, first, the same *J* value from various prevalence scenarios can correspond to different values of $\hat{\alpha }$, and, second, that different *J* values can correspond to the same value of $\hat{\alpha }$.

Prior studies recognizing the sensitivity of similarity indices to species richness have proposed various remedies (*22*–*25*, *30*). One robust-seeming solution is the standardization of the indices in which the difference between the raw value and expectation of the null model is divided by the SD of the null (see #3 under the “Roadmap to the reliable analysis of co-occurrence data” section) (*19*). By analyzing our simulated data with this approach, we show that the standardized Jaccard’s index correctly centers the value of zero at the center of null, as expected for a reliable statistic (section S2 and fig. S1, first column), whereas, for the unreliable raw Jaccard’s index, the center of the cumulative distribution function maps to various values of Jaccard’s index (Fig. 1D). However, the standardized Jaccard’s index still presents two problems as a reliable metric. First, for a given scenario of prevalence, the distribution is not symmetric. This means that values equidistant from the center of the null in opposite directions (e.g., 2 versus −2) indicate different strengths of positive and negative association. Second, across the examples of prevalences, a given distance below the center of the null distribution can mean different degrees of negative association. As a result, standardized *J* results in overprediction or underprediction of the association depending on where the value falls in the null distribution and what the prevalence is (section S2 and fig. S1, third column). In contrast, the cumulative probability distribution of our metric alpha is completely free from these problems (section S2 and fig. S1, second column).

## RESULTS AND DISCUSSION

Below, we reinterpret three high-profile studies of important topics in biogeography, antibiotic resistance, and disease physiology. Each study can be viewed using the classic balls-in-boxes analogy of the hypergeometric and extended hypergeometric distributions (section S3), and each used the Jaccard index in its analyses. The original conclusions of these studies were biologically unexpected, but we show that these interpretations are driven by the sensitivity of the Jaccard index to prevalence. Our application of the affinity metric $\hat{\alpha }$ in reanalyses of the original datasets yields results that contradict the published findings but make more sense biologically.

### Biogeography: Temporal and spatial beta diversity in a Mediterranean archipelago

Beta diversity quantifies the diversity of ecological communities across space (*31*) or compositional change in a single community over time (*7*, *12*). High beta diversity values correspond to high values of species turnover (across space) or high levels of local change (over time). Chiarucci *et al.* (*8*) analyzed floristic composition data of 16 islands of the Tuscan Archipelago, Italy, in two periods, 1830–1950 and 1951–2015, applying the Jaccard index to analyze spatial and temporal beta diversity for a dataset of 10,892 occurrence records across 1831 species. We reanalyzed those data using the affinity model and compared them with Jaccard results. In the setting of temporal beta diversity, the idealized “boxes” are species, cross-classified by “balls” (whose color denotes one of two times) marking the presence/absence of each species on a specific island at a specific time.

The equilibrium theory of island biogeography (*32*) predicts small well-connected islands to have more stable ecological communities than small isolated ones. Similarly, large islands should have more stable communities than small ones. Chiarucci *et al.* (*8*) obtained results opposite to these expectations when they used Jaccard to compare floristic compositions of the islands in the two time periods. We reanalyzed their data using affinity and obtained results that confirmed rather than challenged predictions from island biogeography.

**Fig. 3.**
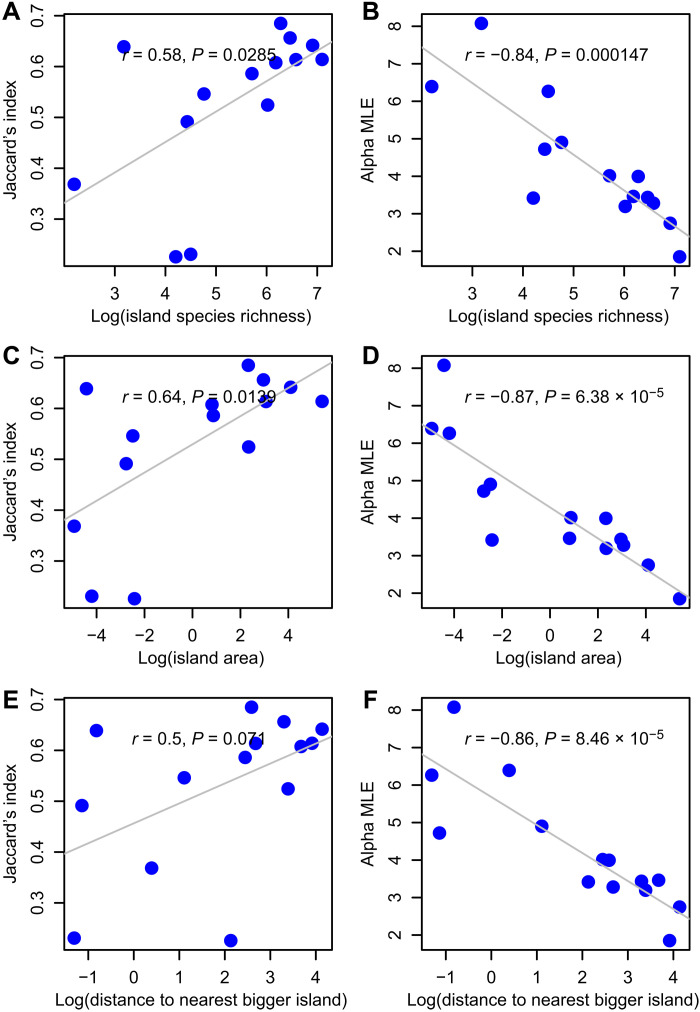
Temporal beta diversity in a Mediterranean archipelago using *J* and $\hat{\alpha }$. Across islands, temporal beta diversity between the two time periods exhibited an unexpected positive log-linear relationship with species richness using *J* (**A**), but a negative log-linear relationship using $\hat{\alpha }$ (**B**). Similar differences in trend appeared for island area (**C** and **D**) and island isolation (**E** and **F**).

Why the difference? The Jaccard and affinity metrics exhibit opposing log-linear relationships as functions of species richness (Fig. 3, A and B), island area (Fig. 3, C and D), and island isolation (Fig. 3, E and F). Hence, inferences drawn about how richness, area, or isolation affects temporal beta diversity change markedly solely on the basis of the metric used. Jaccard is positively biased for species pairs with higher prevalence (section S4 and fig. S2), because high prevalence allows for higher co-occurrence (and higher *J* values) even in null scenarios. Consequently, analyses involving Jaccard predicted positive relationships between temporal beta diversity and species richness or island area because larger islands tended to support more species. In contrast, affinity correctly accounts for differences in prevalence across species pairs and reveals the expected negative relationships between species turnover and richness or area (Fig. 2A).

**Fig. 4.**
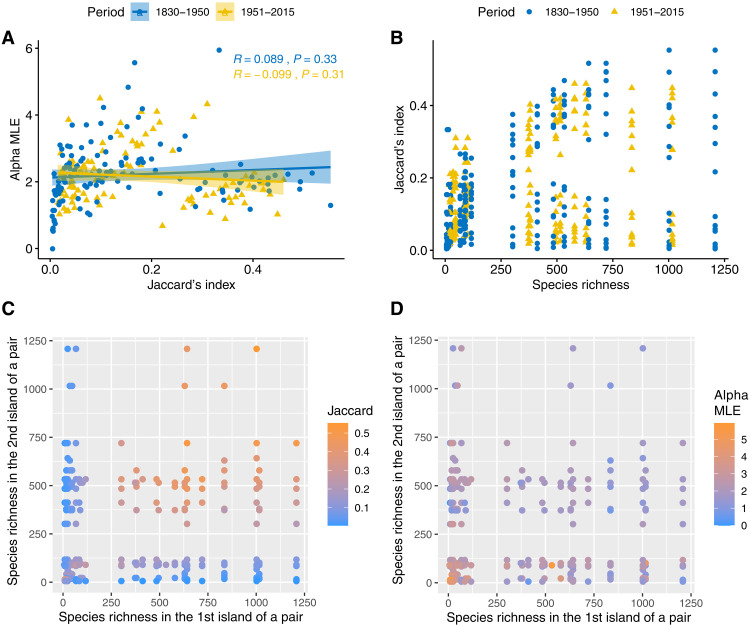
Spatial beta diversity in a Mediterranean archipelago as determined by the traditional *J* and $\hat{\alpha }$. In (**A**), spatial beta diversity between every pair of islands collectively exhibits no relationship between the *J* and $\hat{\alpha }$. In (**B**), *J* diverges to two clusters for species-rich islands. Because *J* is sensitive to prevalence whereas $\hat{\alpha }$ is not, compositional similarity is artificially higher for species-rich islands when calculated using *J* (**C**) (see clustering of orange points in the top right) but not when using $\hat{\alpha }$ (**D**).

Difficulties of interpretation emerge in analyses of spatial beta diversity as well, which, in (*8*), was computed as the similarity in vegetation composition between every possible pair of islands. Here, again, the idealized boxes are species, and the balls (of colors denoting one of a pair of islands) mark the presence/absence of each species on the island. For spatial beta diversity, the Jaccard index and $\hat{\alpha }$ metric have no relationship (Fig. 4A). However, island pairs fall in two clusters (Fig. 4A), resulting from the fact that highly diverse islands (species richness > ~350) have either high or low Jaccard indices (Fig. 4B). When calculated using the Jaccard index, similarity between two species-rich islands is demonstrably higher (Fig. 4C, orange dots) than the similarity between any other combination of island pairs (species-rich versus species-poor, or two species-poor). This is an artifact stemming from the sensitivity of the Jaccard index to prevalence (Fig. 2). However, because $\hat{\alpha }$ is insensitive to prevalence, affinity does not exhibit any systematic pattern with species richness (Fig. 4D), thereby providing a reliable measure of association that can be compared across islands.

### Antibiotic resistance: Reevaluating a cross-resistance interaction network

Patterns of association are critical to understanding the evolution of antibiotic resistance because mutations conferring resistance to specific antibiotics may also afford resistance to others (“cross-resistance”). We reexamined a high-throughput evolutionary experiment that exposed parallel-evolving populations of *Escherichia coli* to different antibiotics (see section S5 for details) (*33*), each of which allowed only resistant mutants to survive. When a particular mutation confers resistance to two antibiotics, the antibiotics are said to “share” that mutation. On the basis of the shared mutations, we mapped the relationships among 12 antibiotics using both the Jaccard index *J* and affinity $\hat{\alpha }$. In this setting, the idealized boxes were mutations, and balls were markers of the presence of mutations in *E. coli* cultured in the presence of each of a pair of antibiotics (the ball “color” being the antibiotic). *J* and $\hat{\alpha }$ are loosely but positively correlated in a nonlinear fashion (fig. S3A), where the antibiotics are associated with very low prevalence of mutations (fig. S3B). We found notable differences in the relationships among antibiotics as judged by *J* and $\hat{\alpha }$ (fig. S3, C and D) including radically different hierarchical clustering of antibiotic similarities (fig. S4, A and B). Specifically, mutation profiles of antibiotic pairs were unlikely to be similar on the basis of *J*, so cross-resistance between members of a pair of antibiotics was judged to be rare. By contrast, many were positively associated on the basis of $\hat{\alpha }$, implying a high likelihood of cross-resistance for pairs of antibiotics that share modes of action (fig. S4B).

In this example, prevalence values were much lower and exhibited relatively modest variation compared to the biogeography example (fig. S2). As a result, the relationship between *J* and $\hat{\alpha }$ is clear and positive. However, *J* remains an inferior metric because it lacks a consistent center, making it impossible to distinguish positive cross-resistance associations from negative ones on the basis of the index alone. In contrast, $\hat{\alpha }$ has a constant center, making it clear which antibiotic cross-resistances are positive versus negative, regardless of prevalence.

### Disease physiology: Biomarkers of oxidative stress in a human disease network

Metrics of association whose values are sensitive to prevalence can also confound understanding of disease physiology. We reanalyzed data for 20 common biomarkers of oxidative stress observed in 34 human diseases (*34*), computing the similarity of disease pairs as a function of shared and unshared biomarkers (see section S6 for details). The idealized boxes in this example are biomarkers for oxidative stress: Balls are indicators that a specific disease shows stress at a specific biomarker, and ball colors are labels for the members of a pair of diseases. *J* and $\hat{\alpha }$ were loosely but positively related over a narrow range of *J* values (fig. S5A), with several diseases having relatively high prevalence of biomarkers (fig. S5B). Most disease pairs showed a weak dissimilarity using *J* (fig. S5C) but a notable mix of positive and negative relationships using $\hat{\alpha }$ (fig. S5D). Again, the hierarchical clustering of disease pairs was radically different for Jaccard and affinity, with affinity suggesting clear sets of diseases that may share underlying mechanisms (fig. S6).

This third example falls between the biogeography and cross-resistance examples in terms of prevalence values. Even so, the Jaccard-affinity differences are marked and lead to radically different mappings of disease pairs with regard to the degree to which they shared 20 biomarkers of oxidative stress. Hierarchical relationships among disease pairs likewise varied greatly, altering inference regarding shared physiological mechanisms proxied by shared biomarkers.

To conclude, our metric of association for binary co-occurrence data effectively groups similar species-pair associations. This grouping is achieved by parameterizing the non-null associations and developing an interpretable degree of association that is insensitive to prevalence. These statistical developments were necessary because widely used measures of co-occurrence suffer from a sensitivity to prevalence that invalidates their use in comparative contexts. Given that the Jaccard, Sørensen-Dice, and Simpson indices are the most common metrics of association in co-occurrence analysis (*18*), it is hard to estimate how much impact their statistical unreliability has had on scientific studies. Google Scholar returns 184,750 results for “Jaccard” when the term is excluded from author names. *Nature* has published 63 papers between 2000 and 2020 using the Jaccard index for similarity (table S1). Given that the Jaccard, Sørensen-Dice, and Simpson indices, as well as ~80 other similar indices (*18*, *19*, *35*, *36*), all use various algebraic transformations of prevalence and copresence without any reference to probability distribution, the sensitivity to prevalence is likely a pervasive problem in analyses of co-occurrence and association. The problems emerge because these indices all assume a fixed range for the metric irrespective of prevalence, whereas the appropriate hypergeometric distribution in the null model has a prevalence-specific range. By contrast, the metric of affinity we developed emerged from a distributional family extending and parameterizing departures from the null hypergeometric distribution. The affinity metric assumes fixed marginals as expected in the scenarios of co-occurrence analysis and remains insensitive to prevalence, allowing it to correctly characterize positive and negative associations across the full spectrum of prevalence values. This fundamentally alters the inferences emerging from diverse biological studies.

## MATERIALS AND METHODS

The abstract context of this paper is a two by two table of counts, tallies of separate and joint occurrences for two types of entities classified into a pair of exhaustive and mutually exclusive categories. Under reasonable assumptions, the probability distribution for the count of co-occurrences is derived mathematically.

The “entities,” “types,” and “categories” can be defined in different ways in different scientific contexts. Entities play the role of balls of two types *A* and *B* that referred to as colors; each ball has a unique label corresponding to its assignment to one of a specified finite set of *N* categories or boxes. In each scientific application, the numbers of balls of the respective colors *A* and *B* are fixed and denoted *m_A_* and *m_B_*. Each of the *m_A_* + *m_B_* balls is assigned to one and only one box, and each box may contain, at most, one ball of each color. Each box may contain either no balls, a single ball of color *A*, a single ball of color *B*, or balls of both colors *A* and *B*. The quantity of interest is the “co-occurrence” count *X* = *a* of the boxes containing balls of both colors. The two by two table in Fig. 1A summarizes that *a* boxes contain balls of both colors, *c* = *m_A_* − *a* boxes contain single *A*-colored balls, *b* = *m_B_* − *a* boxes contain single balls of color *B*, and *d* = *N* − *m_A_* − *m_B_* + *a* boxes contain no balls. Thus, with *N*, *m_A_*, and *m_B_* fixed, the allowed range for the number *a* of co-occurrences is the interval of non-negative integers from max(*m_A_* + *m_B_* − *N*,0) to min(*m_A_*, *m_B_*). We assume from now on that *N*, *m_A_*, and *m_B_* are fixed, with 0 ≤ min (*m_A_*, *m_B_*) ≤ max (*m_A_*, *m_B_*) ≤ *N*.

### Null and affinity model distribution of co-occurrence count

When a probabilistic mechanism governs the assortment of balls into boxes, the co-occurrence count *X* becomes a random variable with possible values ranging from max(*m_A_* + *m_B_* − *N*,0) to min(*m_A_*, *m_B_*), inclusive. If the balls of each color are randomly distributed into boxes, independently across colors, then the probability distribution of *X* is hypergeometric(*N*, *m_A_*, *m_B_*) with the probability mass function given in Eq. 1. This fact, known to Huygens in the 17th century (*1*) and used by Fisher (*5*) in constructing formal statistical hypothesis tests of “no association” between the mechanisms sorting balls of the two colors *A* and *B* into boxes, can be proved as follows.

There are $\left(\frac{N}{m_{A}}\right)$ equiprobable configurations of *m_A_* balls of color *A* in *N* boxes, where “configuration” means a list of which boxes are occupied (by a ball of color *A*) and which are not. Similarly, there are $\left(\frac{N}{m_{B}}\right)$ equiprobable configurations of *m_B_* balls of color *B* in the *N* boxes. For each configuration (denoted *C_A_*) of balls of color *A*, the configurations of balls of color *B* with the additional property that *X* = *k* is the number of boxes containing a ball of both colors can be described uniquely as the set of *k* balls colored *B* falling in the *m_A_* boxes containing balls colored *A*, together with the set of *m_B_* − *k* balls colored *B* falling in the *N* − *m_A_* boxes not containing balls colored *A*. The conditional probability given *C_A_* that the *B*-colored balls fall in such a way that *X* = *k* is therefore equal to the count $\left(\frac{m_{A}}{k})∙(\frac{N-m_{A}}{m_{B}-k}\right)$ of *B* ball configurations satisfying *X* = *k*, divided by the total count $\left(\frac{N}{m_{B}}\right)$ of equiprobable configurations of balls colored *B* in boxes. Because this conditional probability *P*(*X* = *k*∣*C_A_*) = *q_k_* is the same for all possible configurations *C_A_* of *A*-colored balls, it is also the unconditional probability

$$\begin{array}{cc} P(X=k) & =\sum_{C_{A}}P([X=k]\cap C_{A})=\sum_{C_{A}}P(X=k|C_{A})\;P(C_{A})\\ & =\sum_{C_{A}}q_{k}P(C_{A})=q_{k} \end{array}$$ (4)

The proof argument just concluded in Eq. 4 was conditional on the configuration *C_A_* of balls colored *A* into boxes and did not anywhere use the assumption that the $\left(\frac{N}{m_{A}}\right)$ distinct configurations of *A*-colored balls all had the same probability. Therefore, we have proved:

**Proposition 1** *(null hypothesis distribution)* *. If the numbers m_A_*, *m_B_*, *and N are fixed and the m_A_ balls colored A are placed in boxes (with at most one ball to a box) in any way whatever, and, given the placement of the A-colored balls, the m_B_ balls colored B are placed equiprobably in the N boxes, then the probability distribution of the count X of boxes containing balls of both colors is hypergeometric* (*N*, *m_A_*, *m_B_*).

Proposition 1 expresses the unique choice for the reference distribution of the co-occurrence count *X* under the null hypothesis that the mechanisms for *A*- and *B*-colored balls assorting themselves into boxes are independent. These hypothesis tests were formulated in (*5*). However, either to compute power and requisite sample sizes or to quantify appropriately the degree of association in data examples where association is clearly non-null, it is helpful to define a parametric family of alternative distributions for the observed co-occurrence count *X.* This was done first by Fisher in (*6*), through the “Fisher noncentral hypergeometric distribution,” later called “extended hypergeometric” in (*29*), in the following construction that constitutes the affinity model.

Suppose that *m_A_* out of a total of *N* sites contain balls colored *A*, under any mechanism whatever, resulting in configuration *C_A_*. Suppose further that those boxes containing *A* balls are independently chosen with probability *p*_1_ to contain balls colored *B*, while boxes not containing *A* balls are independently chosen with probability *p*_2_ to contain balls colored *B*, with the total number *N_B_* of boxes containing *B*-colored balls conditioned to be *m_B_*. Then, conditionally given the configuration *C_A_* of *A*-colored balls, the probability of exactly *X* = *k* and *N_B_* = *m_B_* is

$$\begin{array}{c} (\frac{m_{A}}{k})\;٠\;p_{1}^{k}\;{\left(1-p_{1}\right)}^{m_{A}-k}\;٠\;(\frac{N-m_{A}}{m_{B}-k})\;{p_{2}}^{m_{B}-k}\;{\left(1-p_{2}\right)}^{N-m_{A}-m_{B}+k}=\\ {\left(1-p_{1}\right)}^{m_{A}}\;{p_{2}}^{m_{B}}\;{\left(1-p_{2}\right)}^{N-m_{A}-m_{B}}\;٠\;(\frac{m_{A}}{k})\;(\frac{N-m_{A}}{m_{B}-k})\;{\left(\frac{p_{1}(1-p_{2})}{p_{2}(1-p_{1})}\right)}^{k} \end{array}$$ (5)

Therefore, *P*(*N_B_* = *m_B_*∣*C_A_*) is equal to the sum over *k* of Eq. 5. Next, letting α = log (*p*_1_(1 − *p*_2_)/*p*_2_(1 − *p*_1_)), we find for max(*m_A_* + *m_B_* − *N*,0) ≤ *k* ≤ min (*m_A_*, *m_B_*)

$$P(X=k|C_{A},N_{B}=m_{B})=\frac{P(X=k,N_{B}=m_{B}\;|\;C_{A})}{P(N_{B}=m_{B}\;|\;C_{A})}$$ (6)

is equal to the expression in Eq. 5 divided by the sum over *k* of the expression in Eq. 5. This ratio is seen to depend on *p*_1_ and *p*_2_ only through α and to be equal to the extended hypergeometric probability mass function in Eq. 3, with parameters *N*, *m_A_*, *m_B_*, and α. Just as in Eq. 4, the fact that the conditional probability in Eq. 6 is the same for all configurations *C_A_* of *A*-colored balls implies that it is also equal to *P*(*X* = *k*∣*N_B_* = *m_B_*). This probability mass function is therefore the unconditional mass function of *X* for the specified mechanism of joint selection of boxes to contain the total numbers *m_A_* and *m_B_* of balls of the two colors, and the same mass function in Eq. 3 arises if the roles of the colors *A* and *B* are reversed in the derivation. We summarize the result in a proposition:

**Proposition 2** *(affinity model distribution)* . *Suppose that the numbers m_A_*, *m_B_*, *and N are fixed, and the m_A_ balls colored A are placed in boxes (with at most one ball to each box) in any way whatever, and given the placement of the A-colored balls, balls colored B are placed independently in boxes with probability p*_1_ *for boxes containing A-colored balls and with probability p*_2_ *for boxes not containing A-colored balls. Then, conditionally given that a total of m_B_ balls are placed in boxes, either conditionally given the configuration of the A-colored balls or unconditionally, the distribution of the count X of co-occurrences is extended hypergeometric with parameters N*, *m_A_*, *m_B_, and* α = log (*p*_1_(1 − *p*_2_)/*p*_2_(1 − *p*_1_)).

### ML estimation of α

The statistical parameter α quantifies the degree of preferential selection for balls colored *B* to occupy boxes already occupied by balls colored *A*. For observed *X* = *k* with fixed *N*, *m_A_*, and *m_B_*, we estimate the unknown real parameter value α ∈ ( −∞, ∞ ) by its ML estimator $\hat{\alpha }$ defined as the maximizer with respect to α of the expression in Eq. 3. Because the extended hypergeometric probability mass function in Eq. 3 is a natural exponential family density [(*37*), section 3.4], the likelihood given by Eq. 3 regarded as a function of α is log concave, and the maximizer is the unique solution of the equation *E*_α_(*X*) = *X*, or

$$X=\sum_{k=0}^{m_{A}}\;k\;(\frac{m_{A}}{k})\;(\frac{N-m_{A}}{m_{B}-k})\;\alpha^{k}/\sum_{k=0}^{m_{A}}\;(\frac{m_{A}}{k})\;(\frac{N-m_{A}}{m_{B}-k})\;\alpha^{k}$$ (7)

when a finite solution exists. The log concavity of *P*_α_(*X* = *k*) implies that the function *E*_α_(*X*) is continuous and strictly increasing, so a finite solution of Eq. 7 fails to exist precisely when either *X* = max (*m_A_* + *m_B_* − *N*,0), in which case we can regard $\hat{\alpha }$ as −∞, or when *X* = min (*m_A_*, *m_B_*), in which case we regard $\hat{\alpha }$ as +∞. Thus, for each true value α ∈ ( −∞, ∞ ), the ML estimate $\hat{\alpha }$ is a discrete extended real valued random variable, because it takes values −∞ or ∞ on the respective positive probability events *X* = max (*m_A_* + *m_B_* − *N*,0) and *X* = min (*m_A_*, *m_B_*).

A simple argument based on Jeffreys’ beta(½, ½) prior distribution for unknown binomial success probabilities allows the conclusion that the maximum estimated absolute value for the binomial log odds should be no greater than log(2*N*) based on *N* Bernoulli trials. Because the log affinity α is best understood as a log odds ratio or a difference between two log odds, it follows that 2 log (2*N*) = log (4*N*^2^) is a natural upper bound for estimated α based on *N* sites. Accordingly, we truncate $\hat{\alpha }$to ± log (4*N*^2^) in place of the ±∞ values that arise (for example, in the small-sample calculations of fig. S4A) when the observed co-occurrence counts hit their extreme possible values, respectively, min(*m_A_*, *m_B_*) or max(*m_A_* + *m_B_* − *N*,0). A further indication of the reasonableness of this modification of $\hat{\alpha }$ is that one can prove mathematically, for all max(*m_A_* + *m_B_* − *N*,0) < *X* < min (*m_A_*, *m_B_*), that

$$|\hat{\alpha }|\leq \text{log}(2(N-\text{max}(m_{A},m_{B})-1)∙\text{min}(m_{A},m_{B}))$$ (8)

and this upper bound for noninfinite values of $|\hat{\alpha }|$is evidently ≤ log (2*N*^2^).

Bounds on the moments of *X*, and applications of them to provide approximate confidence intervals for α, can be found in (*29*). Numerical calculations of the extended hypergeometric probability mass function in R can be based on the package BiasedUrn (*38*).

### Centering for Jaccard’s index null distribution

There are some very special *N*, *m_A_*, and *m_B_* configurations with the property under the null hypothesis that the distribution of the Jaccard’s index centers (has approximate modal value) very close to a fixed value such as ½. Although there is no particularly persuasive null value for the mode of the Jaccard’s index, it may be helpful to highlight the arbitrary nature of the prevalences *m_A_* and *m_B_* under which such centering occurs.

The comment that we are making is simple and algebraic: The Jaccard’s index *a*/(*m_A_* + *m_B_* − *a*) is an increasing function of the hypergeometric (*N*, *m_A_*, *m_B_*) co-occurrence count *a* under the null hypothesis α = 0, so it achieves a modal value near a value *h* such as ½ when the hypergeometric mean *m_A_* ∙ *m_B_*/*N* (close to the mode) substituted for *a* makes *a*/(*m_A_* + *m_B_* − *a*) ≈ *h*. This occurs when

$$\frac{m_{A}∙m_{B}/N}{m_{A}+m_{B}-m_{A}m_{B}/N}\approx h\;\text{or}\;\frac{N}{m_{A}}+\frac{N}{m_{B}}\approx \frac{1+h}{h}$$

In the special case where we look for Jaccard’s index centering near *h* = 0.5, we find that this occurs approximately when *N*/*m_A_* + *N*/*m_B_* ≈ 3, and this occurs for example with *N* = 100 and the special (*m_A_*, *m_B_*) pairs (67,65), (56,82), (80,58), (71,64), and (53,91). The point is that reliable Jaccard null distribution centering can occur for very special prevalence pairs, but not in general, while the distribution of $\hat{\alpha }$under the null hypothesis always centers near 0 regardless of the prevalences.

### Data sources

Data for the biogeography example, including the floristic composition and characteristics of 16 islands, are available as online supplement of the original article (*8*). To compute for additional characteristics of the islands and island pairs, we obtained spatial polygons representing the boundary of the islands from the author of the original publication (*8*) and from Global Administrative Areas database (www.gadm.org; downloaded 26 April 2019). The authors of the original publications provided the datasets for our analyses of antibiotic resistance (*10*, *34*) and diseases physiology (*33*).

### R package

The code necessary to reproduce the analyses in this paper is available as R script in the “Auxiliary Supplementary Materials and Other Supporting Files.” In addition, an R package “CooccurrenceAffinity” featuring an expanded set of output for the affinity model (including ML estimates, confidence intervals, and associated graphics) is available (https://github.com/kpmainali/CooccurrenceAffinity).

## Supplementary Materials

### This PDF file includes:

Sections S1 to S6 Tables S1 and S2 Figs. S1 to S6 Legend for Auxiliary Supplementary Materials and Other Supporting Files References

### Other Supplementary Material for this manuscript includes the following:

Auxiliary Supplementary Materials and Other Supporting Files

## Notes

View/request a protocol for this paper from *Bio-protocol*.
