# Supplementary material for: A better index for analysis of co-occurrence and similarity
Source: Sci Adv. 2022 Jan 26;8(4):eabj9204. doi: 10.1126/sciadv.abj9204 (PMC11633102; doi:10.1126/sciadv.abj9204)
Supplement: Supplementary file 1 — Sections S1 to S6 Tables S1 and S2 Figs. S1 to S6 Legend for Auxiliary Supplementary Materials and Other Supporting Files References [file sciadv.abj9204_sm.pdf]

Science Advances
AAAS

Supplementary Materials for

A better index for analysis of co-occurrence and similarity

Kumar P. Mainali*, Eric Slud, Michael C. Singer, William F. Fagan

*Corresponding author. Email: kpmainali@utexas.edu

Published 26 January 2022, *Sci. Adv.* **8**, eabj9204 (2022)
DOI: 10.1126/sciadv.abj9204

The PDF file includes:

Sections S1 to S6
Tables S1 and S2
Figs. S1 to S6
Legend for Auxiliary Supplementary Materials and Other Supporting Files
References

Other Supplementary Material for this manuscript includes the following:

Auxiliary Supplementary Materials and Other Supporting Files

S1. Published papers in some high-profile journals that used the problematic indices of co-occurrence in their analyses

Table S1. The number of studies published in *Nature*, *Science* and *PNAS* between Jan 2000 and May 2020 using Jaccard, Sørensen–Dice or Simpson indices. For the journal *Nature*, we read all the papers for Jaccard index to ensure the indices were being used in those papers. For other indices in the journal *Nature* as well as for all the indices in the journals *Science* and *PNAS*, we counted the number of papers with the respective terms anywhere in the text except in the author field.

	Jaccard	Sørensen–Dice	Simpson
PNAS	217	638	2291
Science	21	59	304
Nature	63	23	164

S2. Comparing the reliability of standardized Jaccard's index and Alpha for estimating co-occurrence association.

Fig. S1. Reliability of standardized Jaccard's index and Alpha for estimating co-occurrence association. Standardized Jaccard's index (left column) does not map to its cumulative probability in the same way for various scenarios of prevalence whereas this problem is absent for Alpha (middle column), resulting in over-estimation and under-estimation by the standardized index (third column). [figure next page]

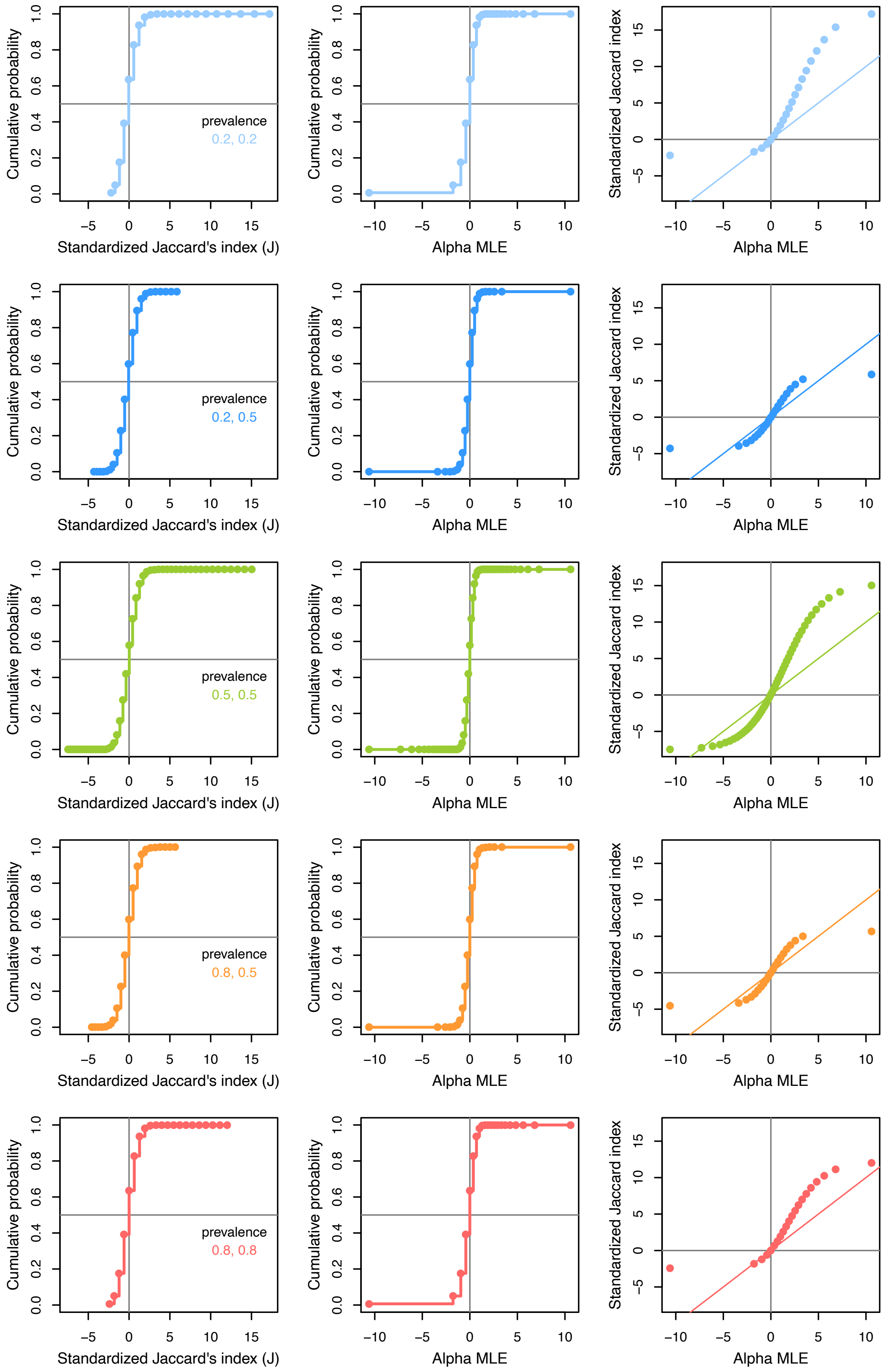
Cumulative probability
Standardized Jaccard's index (J)
Alpha MLE
Standardized Jaccard index
prevalence
0.2, 0.2
0.2, 0.5
0.5, 0.5
0.8, 0.5
0.8, 0.8

S3. Correspondence between example studies and the balls-in-boxes analogy of the Hypergeometric and Extended Hypergeometric Distributions

Table S2. Mapping the Examples into the balls-in-boxes formulation of occupancy and association

Topic/context	Entities = Balls	Colors = Types	Boxes = Categories	Inferred Relationship
Descriptive Ecology	Organisms or individuals in a population	Pair of species	Ecological sites	Habitat similarity between two species
Temporal β-diversity	Selected islands	Pair of times	Species	Community stability of an island
Spatial β-diversity	Islands	Pair of islands	Species	Compositional similarity between islands
Antibiotic Resistance	Cultures in the presence of an antibiotic	Pair of antibiotics	Mutations	Similarity between antibiotics' mutation profiles
Oxidative Stress	Human diseases	Pair of diseases	Biomarkers	Mechanistic similarity between diseases

S4. Extended discussion of the beta diversity example

Fig. S2 presents a histogram of prevalence values for species from the island biogeography example (*8*). The high frequency of species with high prevalence values is largely responsible for the extreme behavior of the traditional Jaccard similarity metric in this example, which contributes to the complete reversal of the relationship between compositional similarity and isolation (Fig. 3).

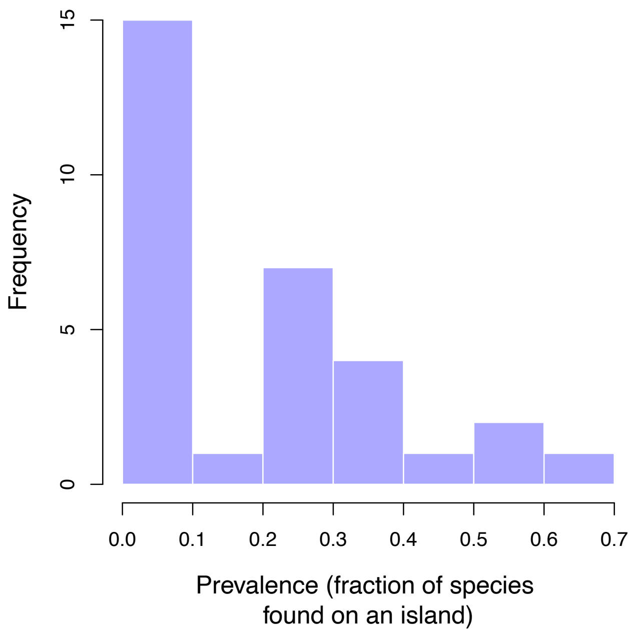

Fig. S2. Prevalence of species richness in the island biogeography examples. For this plot, species richness on an island is defined as species count divided by the total number of species on all islands.

S5. Antibiotic cross-resistance interaction network

To demonstrate the importance of prevalence to metrics of association in a microbiological setting, we reanalyzed data in which parallel evolving cultures of *Escherichia coli* K12 (BW25113) were exposed to each of 12 antibiotics (96 cultures for each antibiotic) in a progressively higher concentration for up to ~336 generations (*33*). Each culture represented an independent evolutionary trajectory with its own population dynamics. The experiment for a given antibiotic ended when at least 10 out of the 96 cultures exhibited growth or when the antibiotic reached its upper limit of solubility. At that point, the authors selected the ten highest density cultures (i.e., the most resistant bacterial populations) and determined their mutation profiles. Based on the binary data of presence/absence of mutations, the authors calculated Jaccard's index between every pair of cultures within or between antibiotics. The authors used the average Jaccard's index (calculated across all parallel cultures for a given antibiotic) to quantify the association of particular mutations with each antibiotic. Because two cultures of a given antibiotic do not necessarily result in the same set of mutations, the association within an antibiotic is not necessarily a theoretical maximum (*9*, *33*). The association between two antibiotics was determined as an average across all pairs of parallel cultures involving those antibiotics. We reanalyzed the data using the affinity model.

For this dataset relating mutations and antibiotics, the Jaccard index and affinity are loosely but positively correlated in a non-linear fashion (Fig. S3a) where the antibiotics are associated with very low prevalence of mutations (fraction of all mutations that are selected by the antibiotic) (Fig. S3b). However, when plotted on identical grids of antibiotics, striking differences emerge between the Jaccard's index and affinity (Figs. S3c,d). In particular, the

entire grid of the Jaccard's index (Fig. S3c) features values confined to the lower half of the range, which was interpreted as antibiotic pairs whose mutations were less likely to be similar. In contrast, $\hat{\alpha}$ shows several antibiotic pairs whose mutations are positively associated; these are particularly prominent along the diagonal of the grid, indicating a positive association between independent cultures of the same antibiotic, which could represent adaptation to the antibiotic (*9*). Differences in association indicated by the Jaccard versus the affinity metric are, however, less dramatic in this example than in the example of beta diversity because the very low prevalence in this example does not inflate Jaccard by the same degree as does the high prevalence of the beta diversity example.

Hierarchical clustering of the antibiotics into functional groups based on their model of action (*9*) also differed depending on which measure of association we used (Fig. S4). The 12 antibiotics grouped into seven functional groups, including five groups with two antibiotics each. Both the Jaccard index and $\hat{\alpha}$ grouped pairs of antibiotics in four of the five functional groups as closely related. However, the two measures of association yielded different dendrogram architectures, suggesting strikingly different relationships among the seven functional groups.

In this example, prevalence values were much lower and exhibited relatively modest variation compared to the biogeography example (Fig. S2). As a result, the relationship between J and $\hat{\alpha}$ is clear and positive. However, J remains an inferior metric because it lacks a consistent center, making it impossible to distinguish positive cross-resistance associations from negative ones on the basis of the index alone. In contrast, $\hat{\alpha}$ has a constant center, making it clear which antibiotic cross-resistances are positive versus negative, regardless of prevalence.

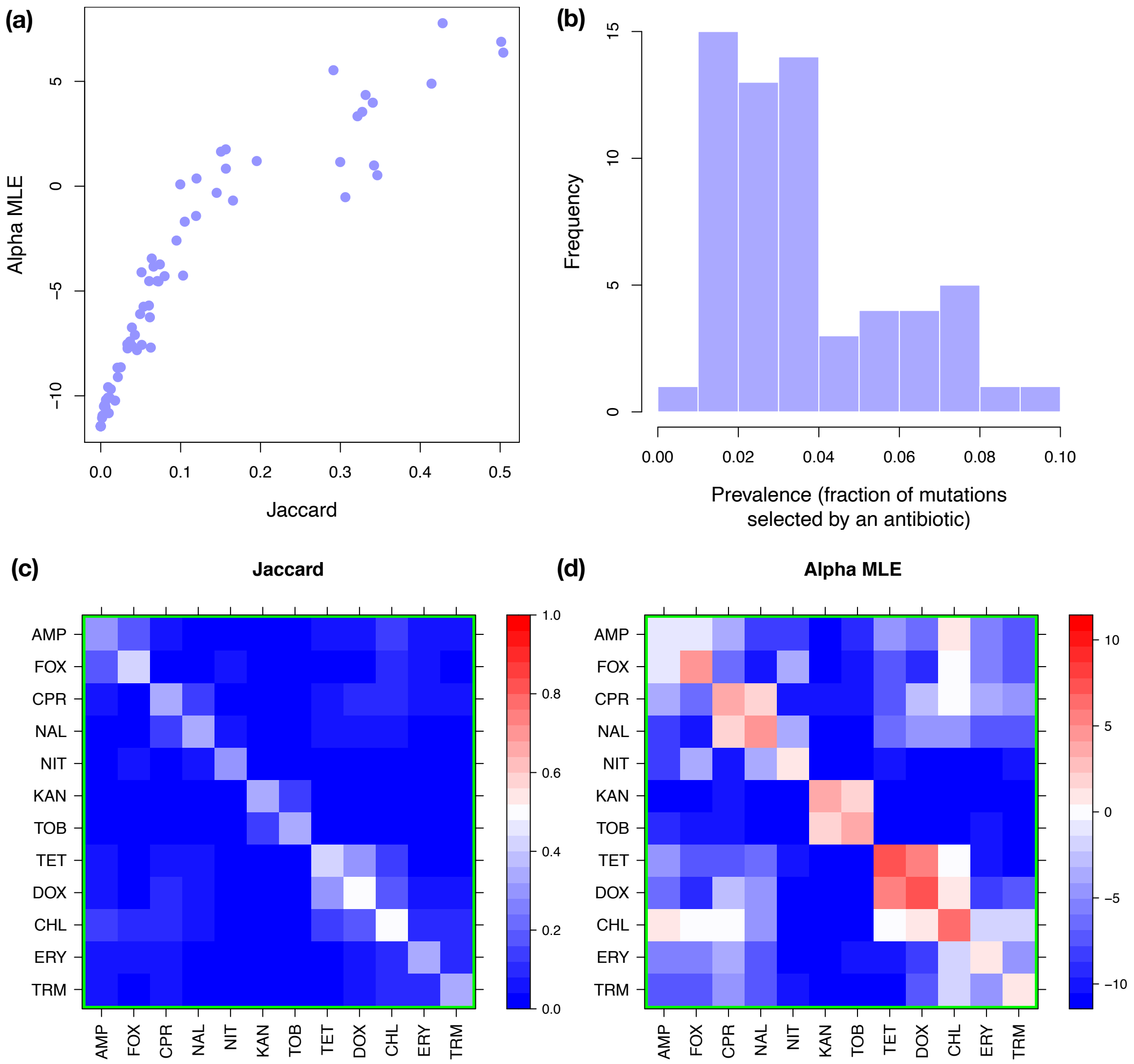

Fig. S3. The Jaccard index and affinity metric as measures of association between the mutation profiles of antibiotic pairs. The Jaccard index and $\hat{\alpha}$ are loosely but positively associated across antibiotic pairs (a) in a case where antibiotics are associated with very low prevalence of mutations (fraction of all mutations that are selected by the antibiotic) (b). Because the Jaccard index is inappropriately sensitive to prevalence of mutations across species (whereas affinity is not sensitive), associations appear as weak / negative on a grid of antibiotic pairs for the Jaccard index (c) but strongly mixed and including some positive associations for $\hat{\alpha}$ (d). Results in (c)

and (d) are plotted on identical antibiotic grids using same color palette that spans the full ranges of the respective metrics. AMP = Ampicillin, FOX = Cefoxitin, CPR = Ciprofloxacin, NAL = Nalidixic Acid, NIT = Nitrofurantoin, KAN = Kanamycin, TOB = Tobramycin, TET = Tetracycline, DOX = Doxycycline, CHL = Chloramphenicol, ERY = Erythromycin, TRM = Trimethoprim.

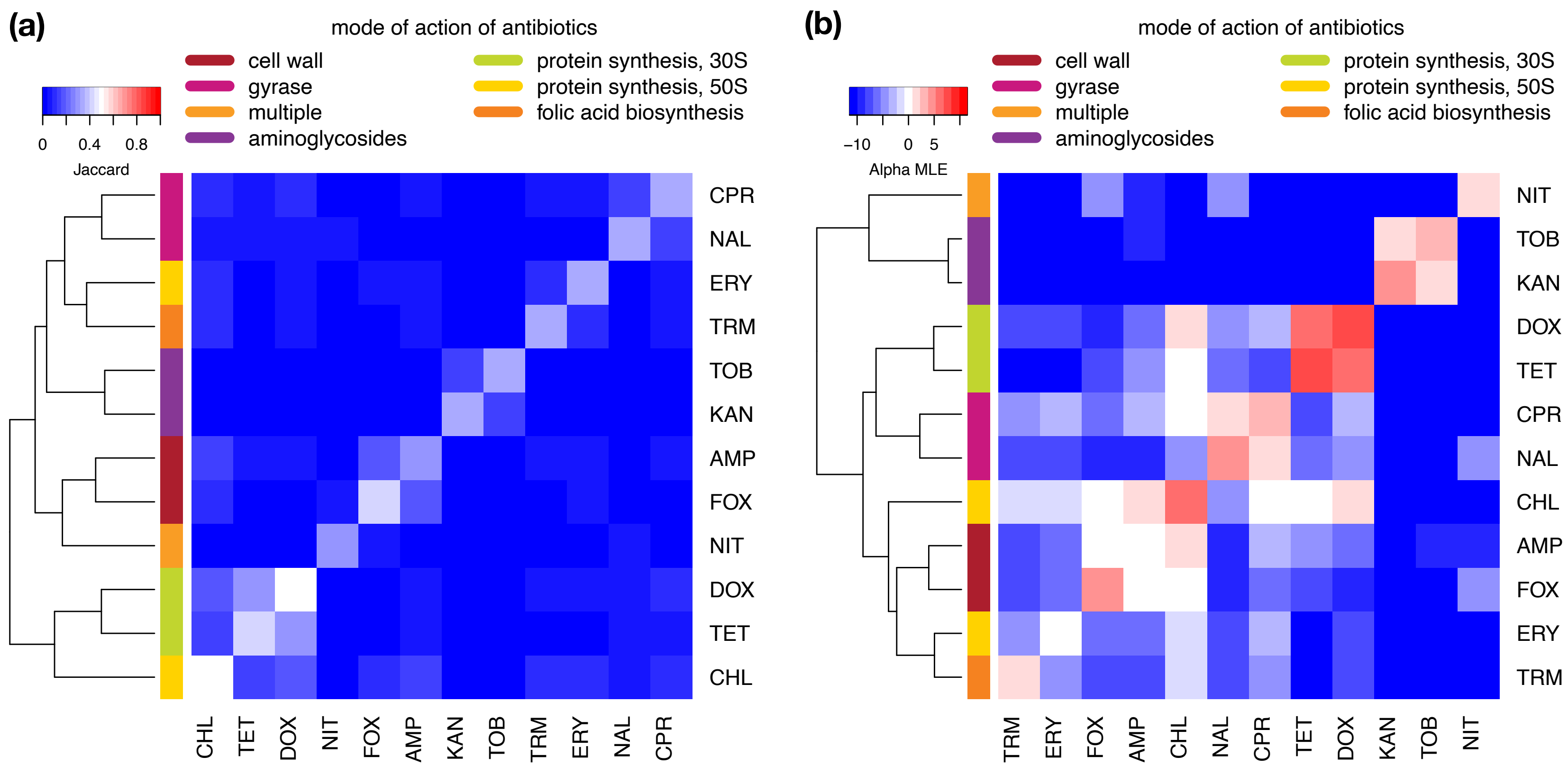

Fig. S4. Antibiotic cross-resistance interaction network as differently quantified by the Jaccard index and affinity. Hierarchical clustering of the Jaccard index (a) and affinity (b) show how differently the antibiotics are sorted.

S6. Human disease network mapped with biomarkers of oxidative stress

As a third example of the importance of excluding effects of prevalence from analyses of associational data, we consider a case from human disease physiology. Many diseases are caused by oxidative stress (*39*) which results from abnormally high levels of reactive oxygen species (*40*). Unfortunately, the very short half-life of reactive oxygen species largely prohibits the measurement of those oxygen species in biological fluids (*41*). However, correlated indicators of oxidative stress can be measured, and these proxies of oxidative stress can be utilized as diagnostic or prognostic indicators. Such proxies – more formally, molecular biomarkers – are substances that, when found outside a normal range, correlate with some pathological process (*10*). We obtained published data (*34*) on 20 common biomarkers of oxidative stress that were observed in 34 diseases. Using the presence/absence state of the biomarkers in each disease, we computed the similarity of each disease-pair using the shared and unshared biomarkers, using both the Jaccard index and affinity.

The Jaccard's index and affinity are loosely but positively related for a narrow range of the Jaccard index, with several pairs securing high affinity for low value of Jaccard index (Fig. S5a). This behavior is explained by the fact that several diseases have relatively high prevalence of biomarkers (Fig. S5b). This confounds inferences about associations between biomarkers and disease to a level intermediate between the beta diversity and antibiotic resistance examples. Most disease-pairs show a weak dissimilarity for the Jaccard index (Fig. S5c). In contrast, the color profile of affinity (Fig. S5d) shows a striking mix of positively and negatively associated disease pairs. A dendrogram of each of these two metrics show that many of the clusters of diseases are similar between the indices with some important differences (Fig. S6).

This third example falls between the biogeography and cross-resistance examples in terms of prevalence values. Even so, the Jaccard-affinity differences are dramatic, and lead to radically different mappings of disease pairs with regard to the degree to which they shared 20 biomarkers of oxidative stress. Hierarchical relationships among disease pairs likewise varied greatly, altering inference regarding shared physiological mechanisms proxied by shared biomarkers.

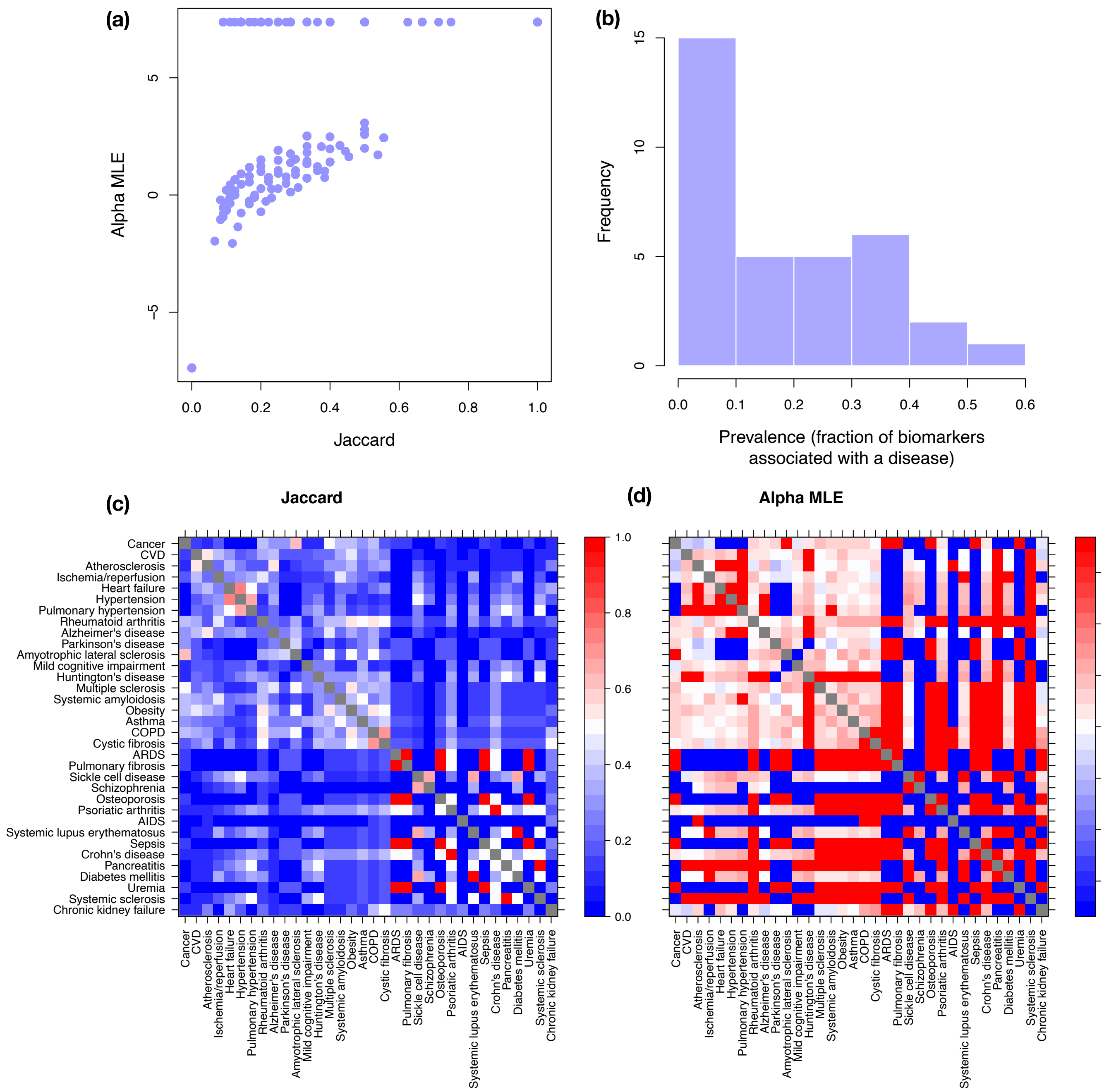

Fig. S5. Associations among 34 human disease pairs mapped according to the degree to which they shared 20 biomarkers of oxidative stress. (a) Scatterplot between Jaccard index and Affinity metric $\hat{\alpha}$, (b) prevalence of biomarkers among diseases, (c) heatmap of Jaccard index between diseases, and (d) heatmap of $\hat{\alpha}$ between diseases. Jaccard index and $\hat{\alpha}$ are plotted on identical disease grids using the same color palette that spans the full ranges of the respective metrics.

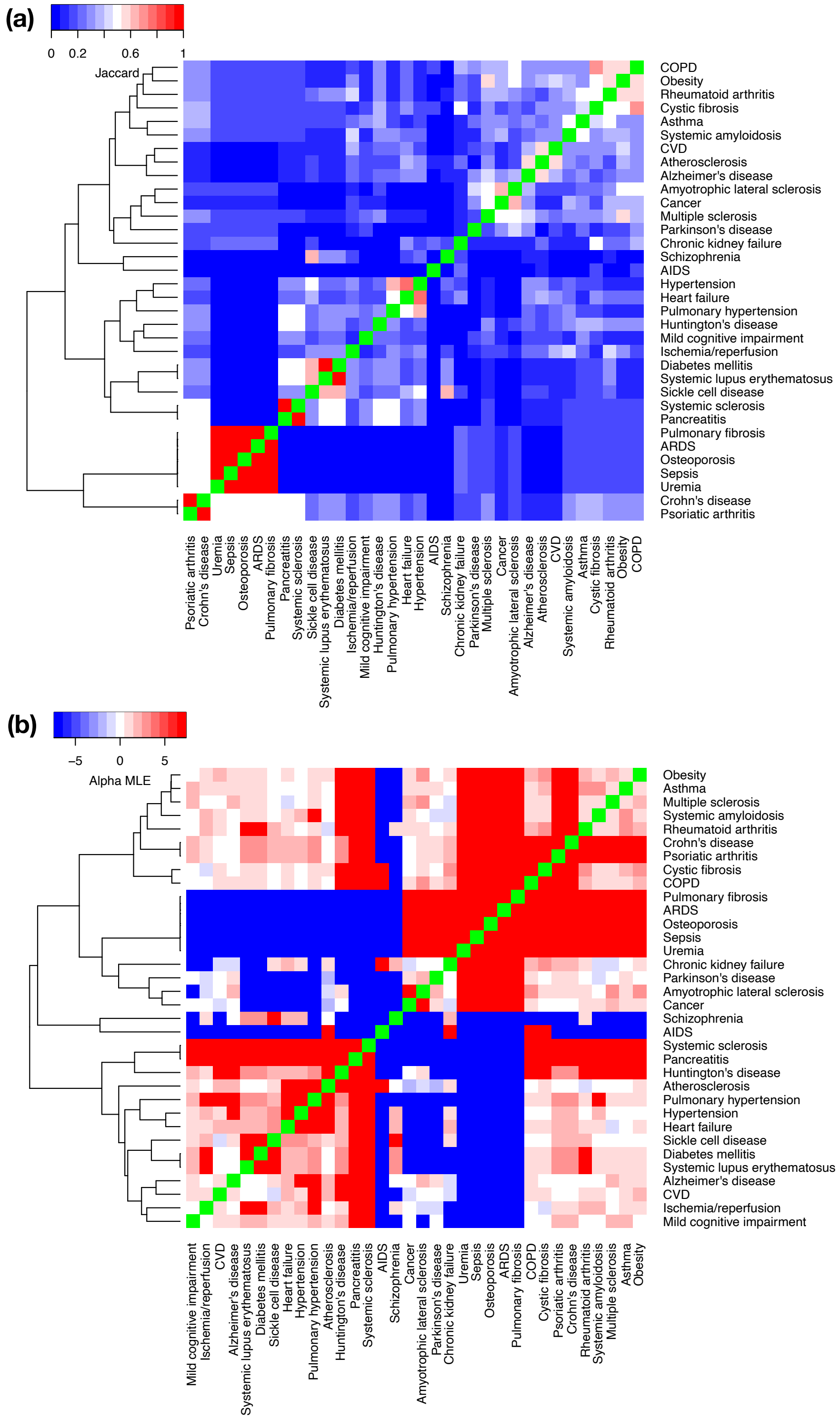

Fig. S6. Hierarchical clustering of disease similarity based on the Jaccard index (a) and affinity (b) exhibits important differences in disease sorting, which alters inference regarding shared physiological mechanisms proxied by shared biomarkers.

Auxiliary Supplementary Materials and Other Supporting Files

A zipped folder with the complete data and **R** code necessary to reproduce the analyses in this paper is available here.

REFERENCES AND NOTES

1. C. Huygens, *Libellus de Ratiociniis in Ludo Aleae. Or, The Value of All Chances in Games of Fortune; Cards, Dice, Wagers, Lotteries, &c. Mathematically Demonstrated* (S. Keimer, London, 1714).

2. S. Stigler, The missing early history of contingency tables. *Ann. la Fac. des Sci. Toulouse Mathématiques*. **11**, 563–573 (2002).

3. L. A. J. Quetelet, *Sur l'Homme et le Développement de ses Facultés, ou Essai de Physique Sociale* (Bachelier, Paris, 1835), vol. 2.

4. K. Pearson, X. On the criterion that a given system of deviations from the probable in the case of a correlated system of variables is such that it can be reasonably supposed to have arisen from random sampling. *Philos. Mag.* **50**, 157–175 (1900).

5. R. A. Fisher, *Statistical Methods for Research Workers* (Oliver and Boyd, Edinburgh and London, ed. 5, 1934).

6. R. A. Fisher, The logic of inductive inference. *J. R. Stat. Soc.* **98**, 39–82 (1935).

7. M. Dornelas, N. J. Gotelli, B. McGill, H. Shimadzu, F. Moyes, C. Sievers, A. E. Magurran, Assemblage time series reveal biodiversity change but not systematic loss. *Science (80-.).* **344**, 296–299 (2014).

8. A. Chiarucci, S. Fattorini, B. Foggi, S. Landi, L. Lazzaro, J. Podani, D. Simberloff, Plant recording across two centuries reveals dramatic changes in species diversity of a Mediterranean archipelago. *Sci. Rep.* **7**, 5415 (2017).

9. V. Lázár, I. Nagy, R. Spohn, B. Csörgő, Á. Györkei, Á. Nyerges, B. Horváth, A. Vörös, R. Busa-Fekete, M. Hrtyan, B. Bogos, O. Méhi, G. Fekete, B. Szappanos, B. Kégl, B. Papp, C. Pál, Genome-wide analysis captures the determinants of the antibiotic cross-resistance interaction network. *Nat. Commun.* **5**, 4352 (2014).

10. P. Ghezzi, K. Davies, A. Delaney, L. Floridi, Theory of signs and statistical approach to big data in assessing the relevance of clinical biomarkers of inflammation and oxidative stress. *Proc. Natl. Acad. Sci.* **115**, 2473–2477(2018).

11. B. R. de Forges, J. A. Koslow, G. C. B. Poore, Diversity and endemism of the benthic seamount fauna in the southwest Pacific. *Nature* **405**, 944 –947 (2000).

12. S. A. Blowes, S. R. Supp, L. H. Antão, A. Bates, H. Bruelheide, J. M. Chase, F. Moyes, A. Magurran, B. McGill, I. H. Myers-Smith M. Winter, A. D. Bjorkman, D. E. Bowler, J. E. K. Byrnes, A. Gonzalez, J. Hines, F. Isbell, H. P. Jones, L. M. Navarro, P. L. Thompson, M. Vellend, C. Waldock, M. Dornelas, The geography of biodiversity change in marine and terrestrial assemblages. *Science* **366**, 339–345 (2019).

13. J. M. Chase, M. A. Leibold, Spatial scale dictates the productivity-biodiversity relationship. *Nature* **416**, 427 (2002), 430.

14. N. J. Gotelli, D. J. McCabe, Species co-occurrence: A meta-analysis of JM Diamond's assembly rules model. *Ecology* **83**, 2091–2096 (2002).

15. J. Cornfield, in *Proceedings of the Third Berkeley Symposium on Mathematical Statistics and Probability* (University of California Press, 1956), vol. 4, pp. 135–148.

16. G. Plata, C. S. Henry, D. Vitkup, Long-term phenotypic evolution of bacteria. *Nature* **517**, 369 (2015), 372.

17. N. A. Crossley, A. Mechelli, P. E. Vértes, T. T. Winton-Brown, A. X. Patel, C. E. Ginestet, P. McGuire, E. T. Bullmore, Cognitive relevance of the community structure of the human brain functional coactivation network. *Proc. Natl. Acad. Sci.* **110**, 11583–11588 (2013).

18. M. E. Hohn, in *Handbook of Mathematical Geosciences*, B. S. Daya Sagar, Q. Cheng, F. Agterberg, Eds. (Springer, 2018), pp. 143–160.

19. P. Keil, Z-scores unite pairwise indices of ecological similarity and association for binary data. *Ecosphere*. **10**, e02933 (2019).

20. J. A. Veech, A probabilistic model for analysing species co-occurrence. *Glob. Ecol. Biogeogr.* **22**, 252–260 (2013).

21. D. M. Griffith, J. A. Veech, C. J. Marsh, Cooccur: Probabilistic species co-occurrence analysis in R. *J. Stat Softw*. **69**, 1–17 (2016).

22. W. Ulrich, A. Baselga, B. Kusumoto, T. Shiono, H. Tuomisto, Y. Kubota, The tangled link between β-and γ-diversity: A Narcissus effect weakens statistical inferences in null model analyses of diversity patterns. *Glob. Ecol. Biogeogr.* **26**, 1–5 (2017).

23. W. Ulrich, Y. Kubota, B. Kusumoto, A. Baselga, H. Tuomisto, N. J. Gotelli, Species richness correlates of raw and standardized co-occurrence metrics. *Glob. Ecol. Biogeogr*. **27**, 395–399 (2018).

24. N. J. Gotelli, W. Ulrich, Statistical challenges in null model analysis. *Oikos* **121**, 171–180 (2012).

25. J. R. Bennett, B. Gilbert, Contrasting beta diversity among regions: How do classical and multivariate approaches compare? *Glob. Ecol. Biogeogr.* **25**, 368–377 (2016).

26. D. I. MacKenzie, J. D. Nichols, J. A. Royle, K. H. Pollock, L. L. Bailey, J. E. Hines, *Occupancy Estimation and Modeling: Inferring Patterns and Dynamics of Species Occurrence* (Elsevier, 2006).

27. Y. M. Bishop, S. E. Fienberg, P. W. Holland, *Discrete Multivariate Analysis: Theory and Practice* (MIT Press, 1975).

28. A. Agresti, *Categorical Data Analysis* (John Wiley & Sons Inc., ed. 3, 2013).

29. W. L. Harkness, Properties of the extended hypergeometric distribution. *Ann. Math. Stat.* **36**, 938–945 (1965).

30. W. Ulrich, N. J. Gotelli, Pattern detection in null model analysis. *Oikos* **122**, 2–18 (2013).

31. R. H. Whittaker, Vegetation of the Siskiyou Mountains, Oregon and California, *Ecol. Monogr.* **30**, 279–338 (1960).

32. R. H. MacArthur, E. O. Wilson, *The Theory of Island Biogeography* (Princeton Univ. Press, 2001), vol. 1.

33. V. Lázár, G. P. Singh, R. Spohn, I. Nagy, B. Horváth, M. Hrtyan, R. Busa-Fekete, B. Bogos, O. Méhi, B. Csörgo, G. Pósfai, G. Fekete, B. Szappanos, B. Kégl, B. Papp, C. Pál, Bacterial evolution of antibiotic hypersensitivity. *Mol. Syst. Biol.* **9** (2013).

34. J. Frijhoff, P. G. Winyard, N. Zarkovic, S. S. Davies, R. Stocker, D. Cheng, A. R. Knight, E. L. Taylor, J. Oettrich, T. Ruskovska, A. C. Gasparovic, A. Cuadrado, D. Weber, H. E. Poulsen, T. Grune, Harald H H W Schmidt, P. Ghezzi, Clinical relevance of biomarkers of oxidative stress. *Antioxid. Redox Signal.* **23**, 1144–1170 (2015).

35. Z. Hubálek, Coefficients of association and similarity, based on binary (presence-absence) data: An evaluation. *Biol. Rev.* **57**, 669–689 (1982).

36. S. H. Hurlbert, A coefficient of interspecific assciation. *Ecology* **50**, 1–9 (1969).

37. G. Casella, R. L. Berger, *Statistical Inference* (Duxbury Pacific Grove, 2002).

38. A. Fog, R Package ‘BiasedUrn’ (2013); https://cran.r-project.org.

39. P. Ghezzi, V. Jaquet, F. Marcucci, H. H. H. W. Schmidt, The oxidative stress theory of disease: Levels of evidence and epistemological aspects. *Br. J. Pharmacol.* **174**, 1784–1796 (2017).

40. H. Sies, Oxidative stress: A concept in redox biology and medicine. *Redox Biol.* **4**, 180–183 (2015).

41. J. P. Kehrer, The Haber-Weiss reaction and mechanisms of toxicity. *Toxicology* **149**, 43–50 (2000).
