# Supplementary figures and images for: A better index for analysis of co-occurrence and similarity
Source: Sci Adv. 2022 Jan 26;8(4):eabj9204. doi: 10.1126/sciadv.abj9204 (PMC11633102; doi:10.1126/sciadv.abj9204)

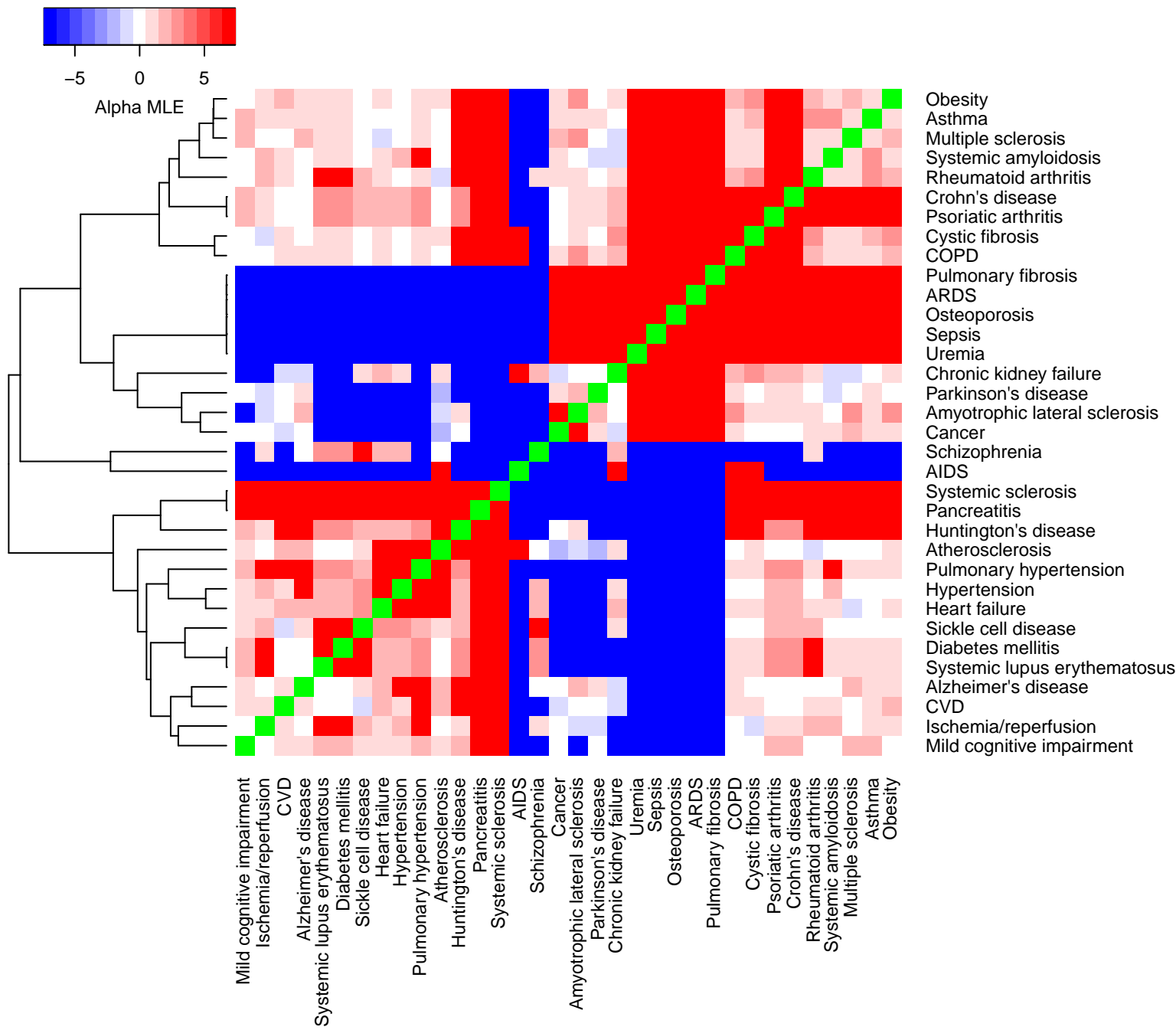

Supplement: Supplementary file 2 — Auxiliary Supplementary Materials and Other Supporting Files [file sciadv.abj9204_auxiliary_supplementary_materials_and_other_supporting_files.zip › codesupplement/plots/Fig S6 - AffinityMLE_completeplot.pdf]

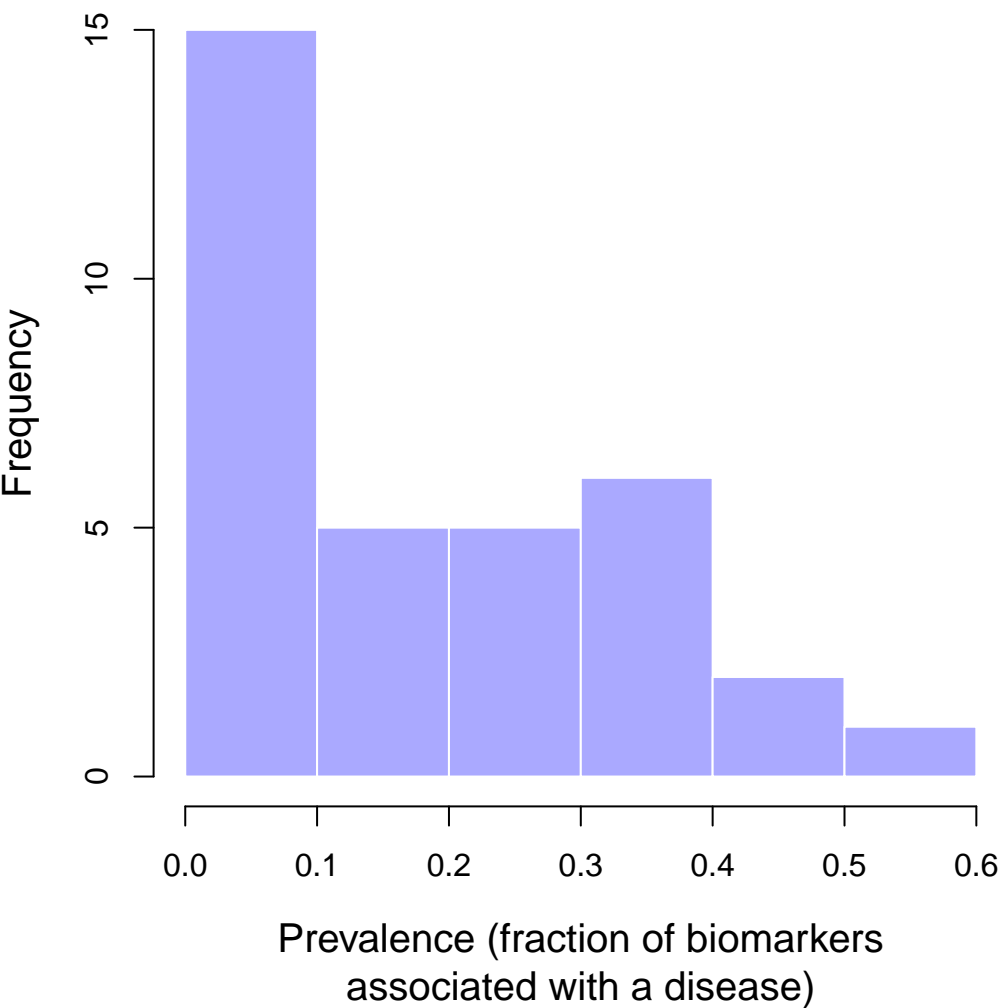

Supplement: Supplementary file 2 — Auxiliary Supplementary Materials and Other Supporting Files [file sciadv.abj9204_auxiliary_supplementary_materials_and_other_supporting_files.zip › codesupplement/plots/Fig S5 - biomarkers hist prevalence.pdf]

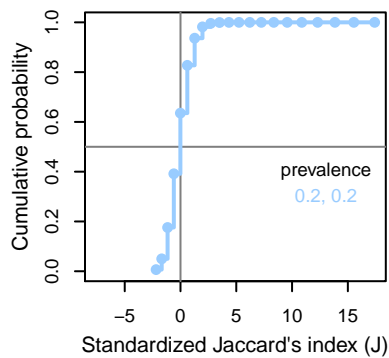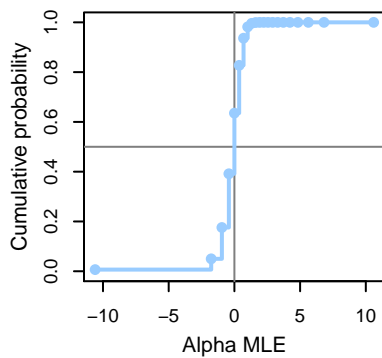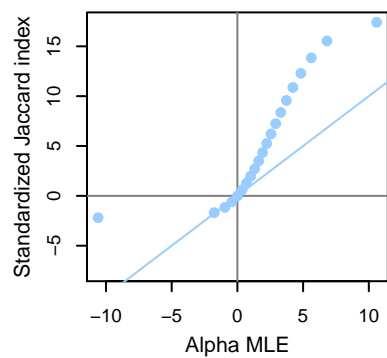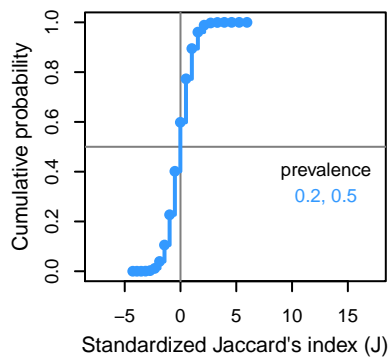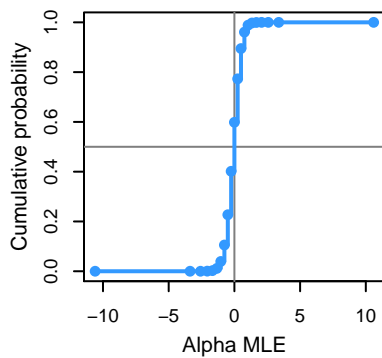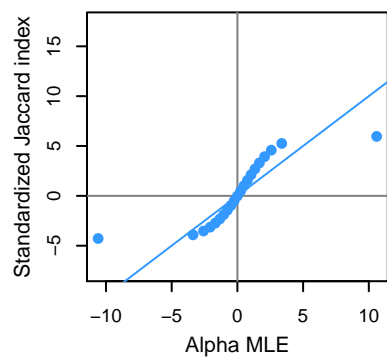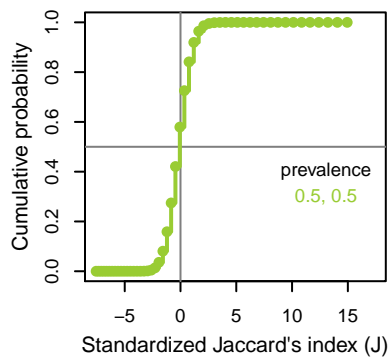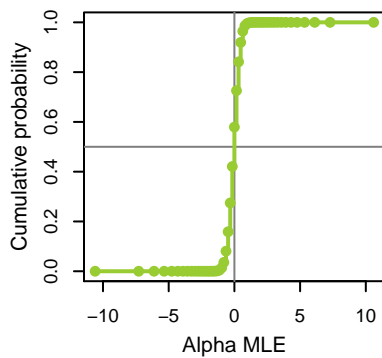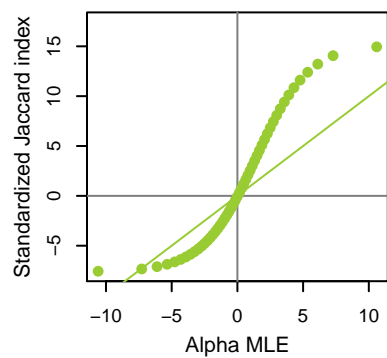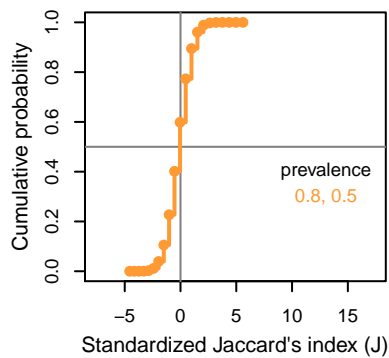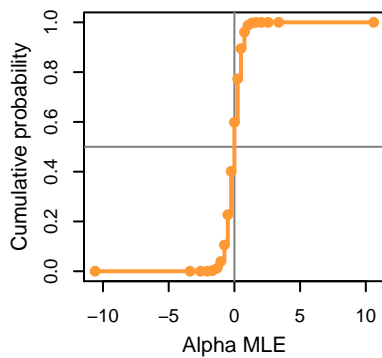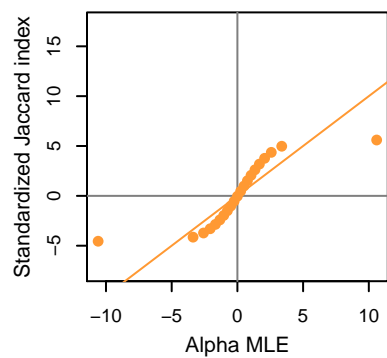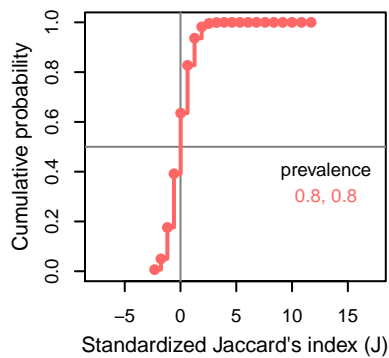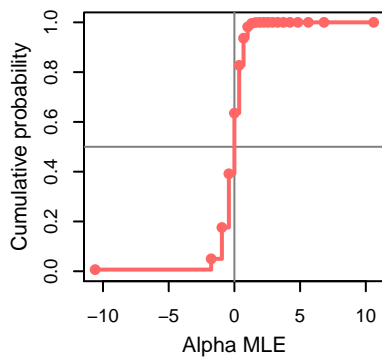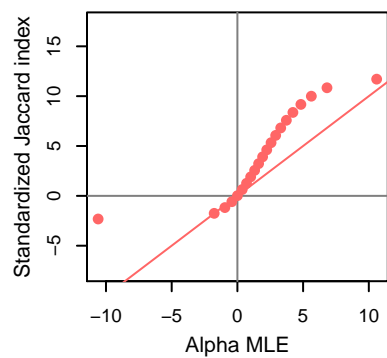

Supplement: Supplementary file 2 — Auxiliary Supplementary Materials and Other Supporting Files [file sciadv.abj9204_auxiliary_supplementary_materials_and_other_supporting_files.zip › codesupplement/plots/Fig S1 - standardized Jaccard by cumulative probability and alpha.pdf]

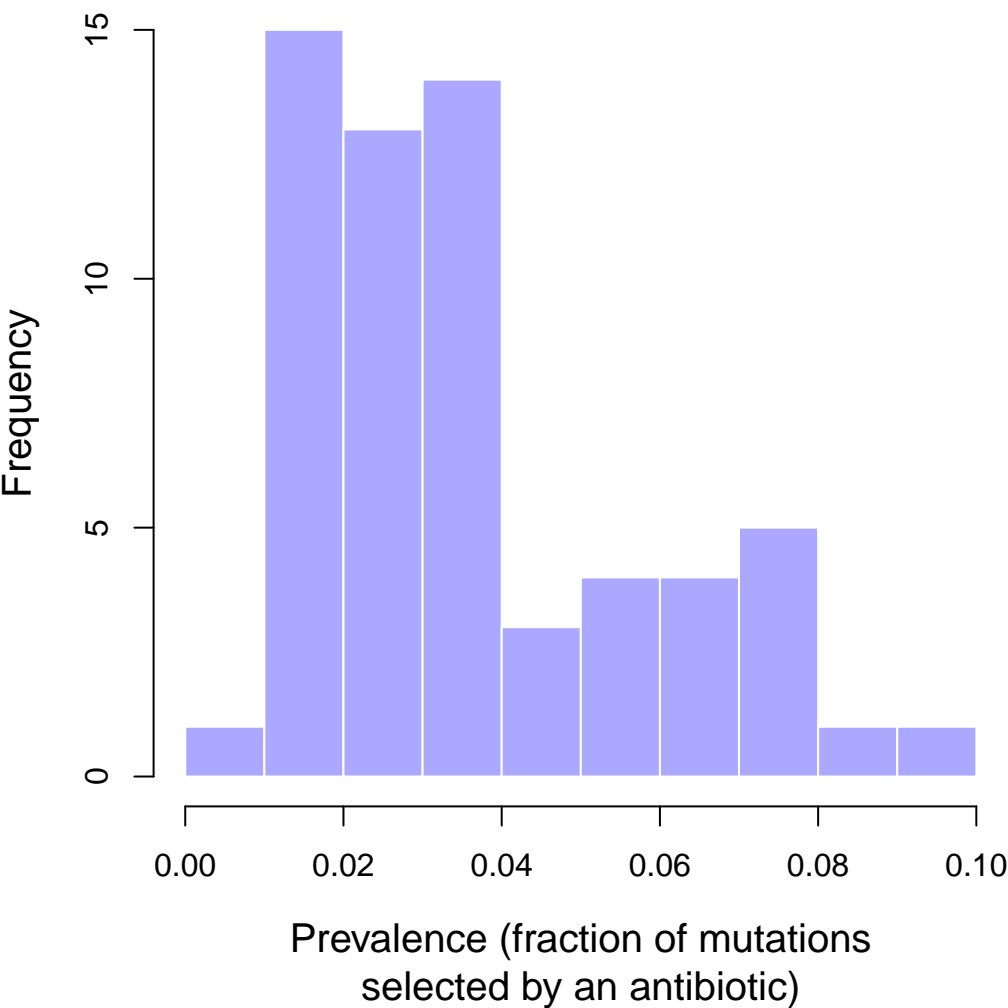

Supplement: Supplementary file 2 — Auxiliary Supplementary Materials and Other Supporting Files [file sciadv.abj9204_auxiliary_supplementary_materials_and_other_supporting_files.zip › codesupplement/plots/Fig S3B - antibiotic hist average prevalence.pdf]

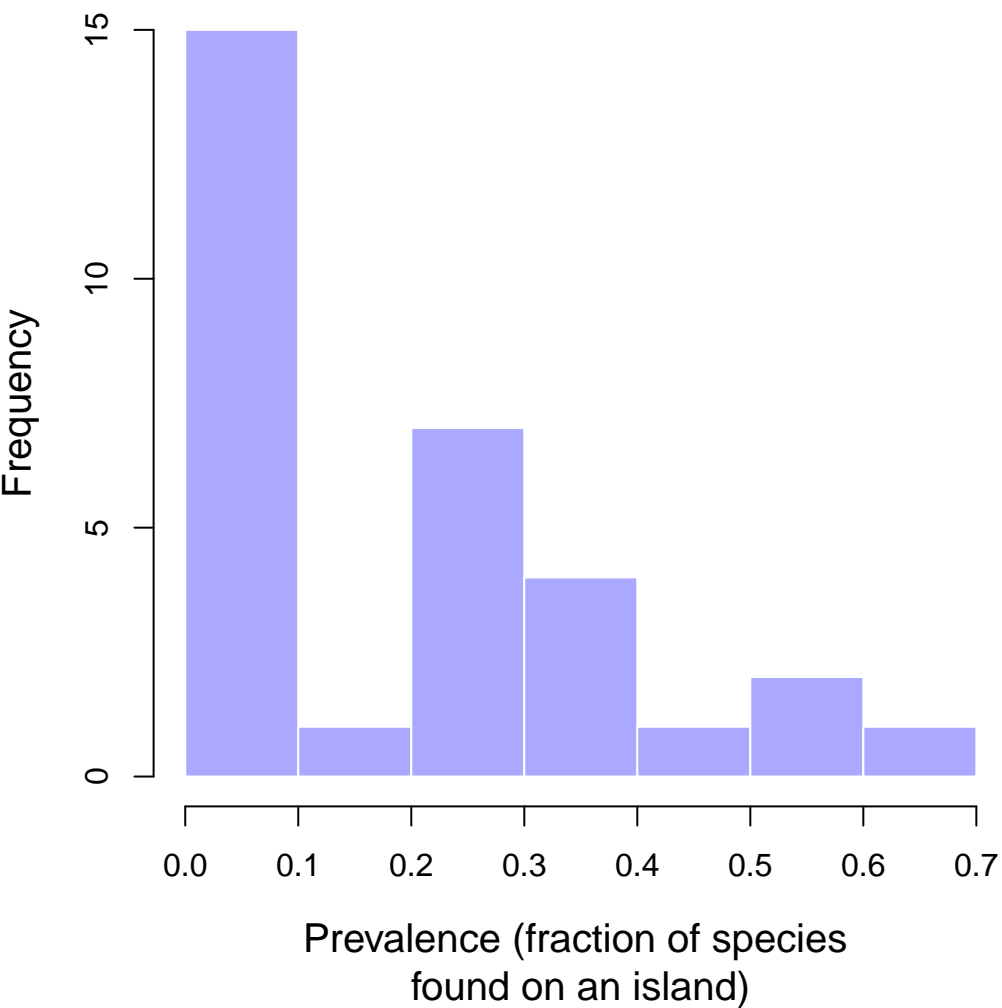

Supplement: Supplementary file 2 — Auxiliary Supplementary Materials and Other Supporting Files [file sciadv.abj9204_auxiliary_supplementary_materials_and_other_supporting_files.zip › codesupplement/plots/Fig S2 - beta diversity species richness prevalence.pdf]

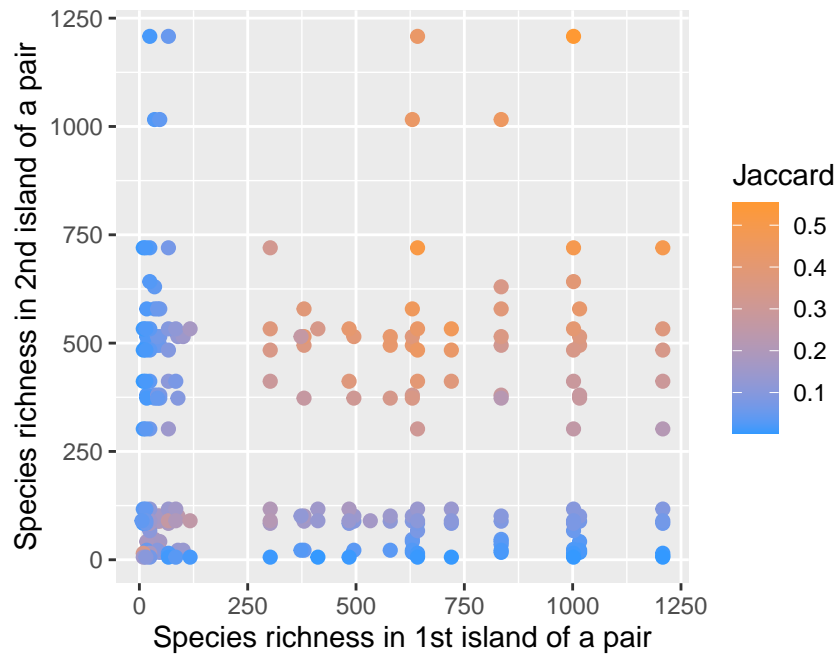

Supplement: Supplementary file 2 — Auxiliary Supplementary Materials and Other Supporting Files [file sciadv.abj9204_auxiliary_supplementary_materials_and_other_supporting_files.zip › codesupplement/plots/Fig 4C - jaccard in island pairs.pdf]

## Jaccard

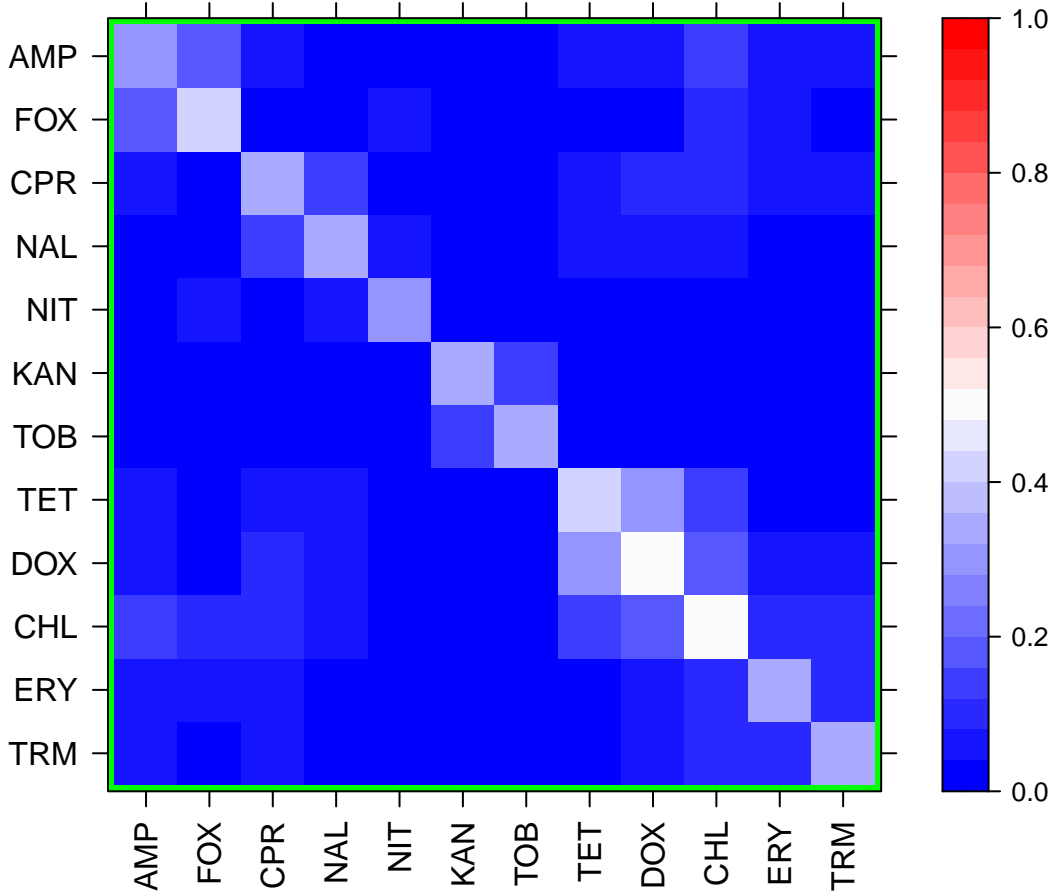

Supplement: Supplementary file 2 — Auxiliary Supplementary Materials and Other Supporting Files [file sciadv.abj9204_auxiliary_supplementary_materials_and_other_supporting_files.zip › codesupplement/plots/Fig S3C - antibiotic_Jaccard_newcolor_levelplot_colorScaled.pdf]

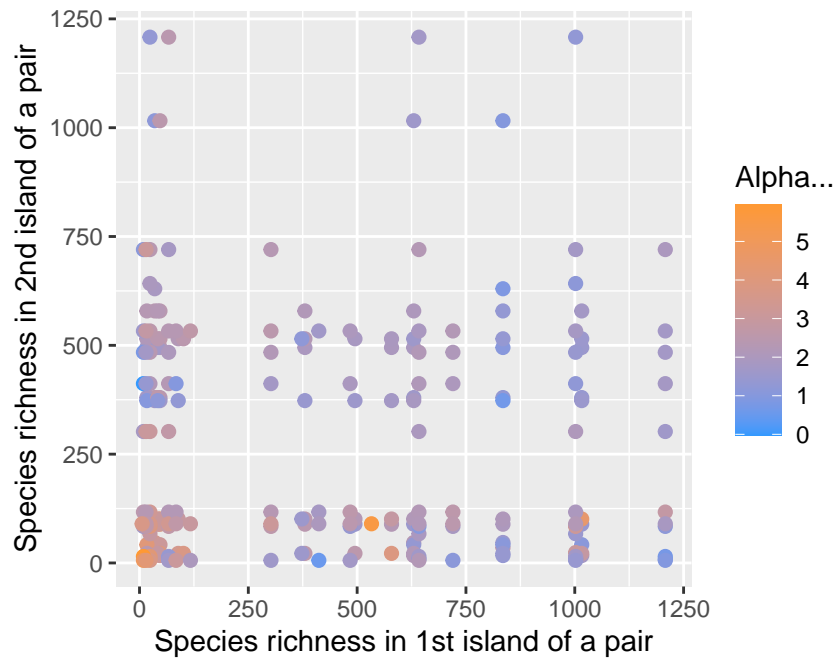

Supplement: Supplementary file 2 — Auxiliary Supplementary Materials and Other Supporting Files [file sciadv.abj9204_auxiliary_supplementary_materials_and_other_supporting_files.zip › codesupplement/plots/Fig 4D - alpha in island pairs.pdf]

period    ● 1830–1950    ▲ 1951–2015

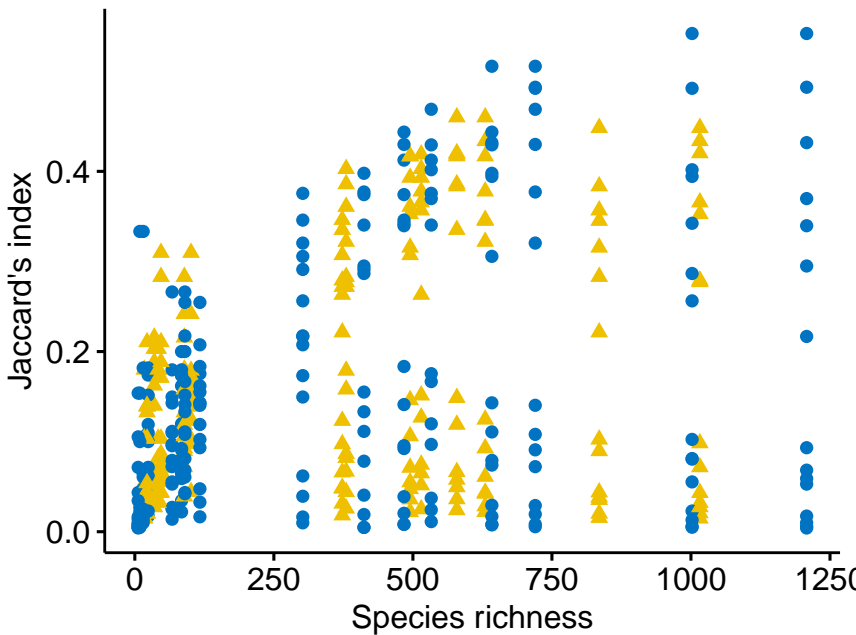

Supplement: Supplementary file 2 — Auxiliary Supplementary Materials and Other Supporting Files [file sciadv.abj9204_auxiliary_supplementary_materials_and_other_supporting_files.zip › codesupplement/plots/Fig 4B - mediterreanean species richness vs jaccard2.pdf]

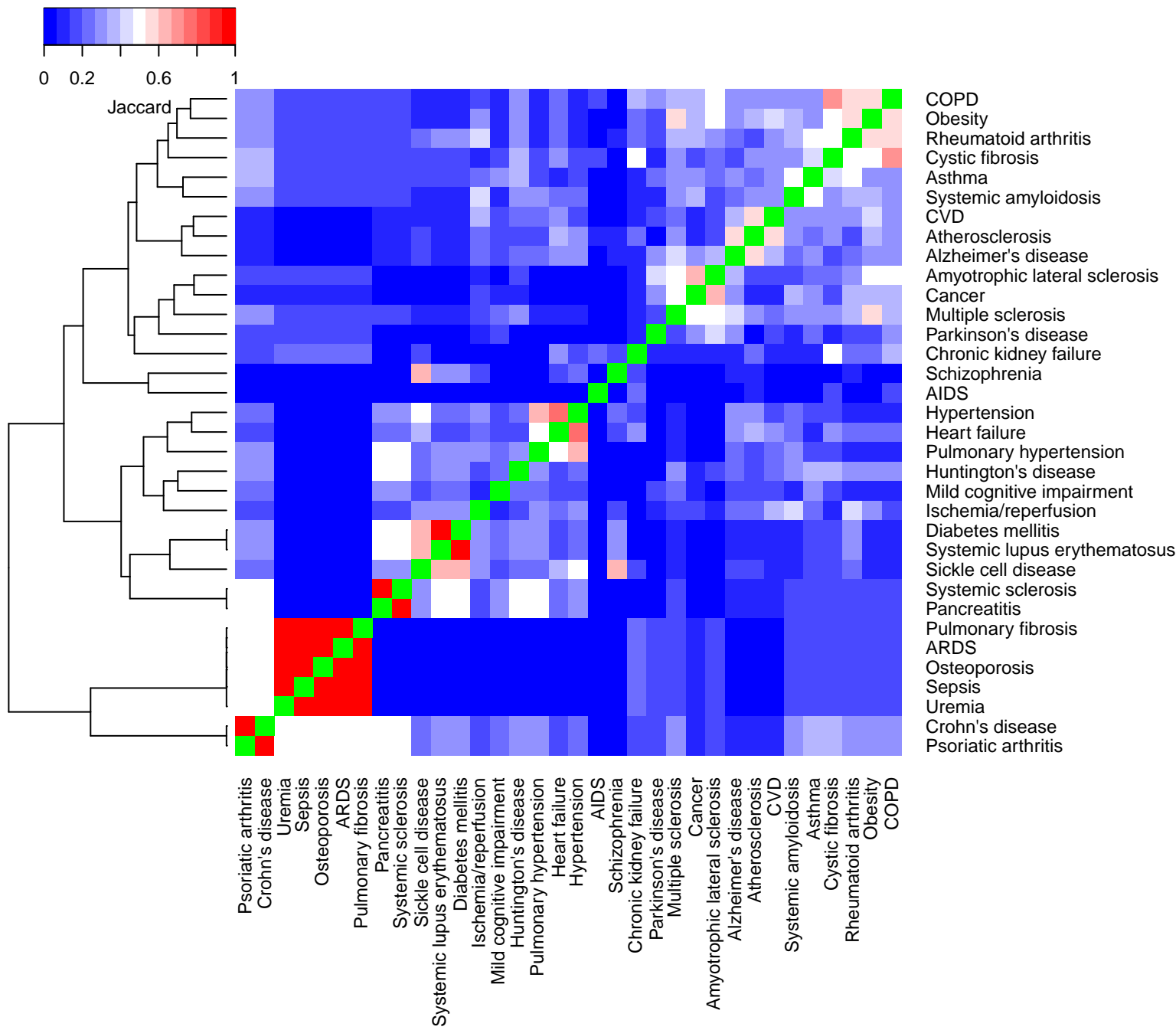

Supplement: Supplementary file 2 — Auxiliary Supplementary Materials and Other Supporting Files [file sciadv.abj9204_auxiliary_supplementary_materials_and_other_supporting_files.zip › codesupplement/plots/Fig S6 - Jaccard_completeplot.pdf]

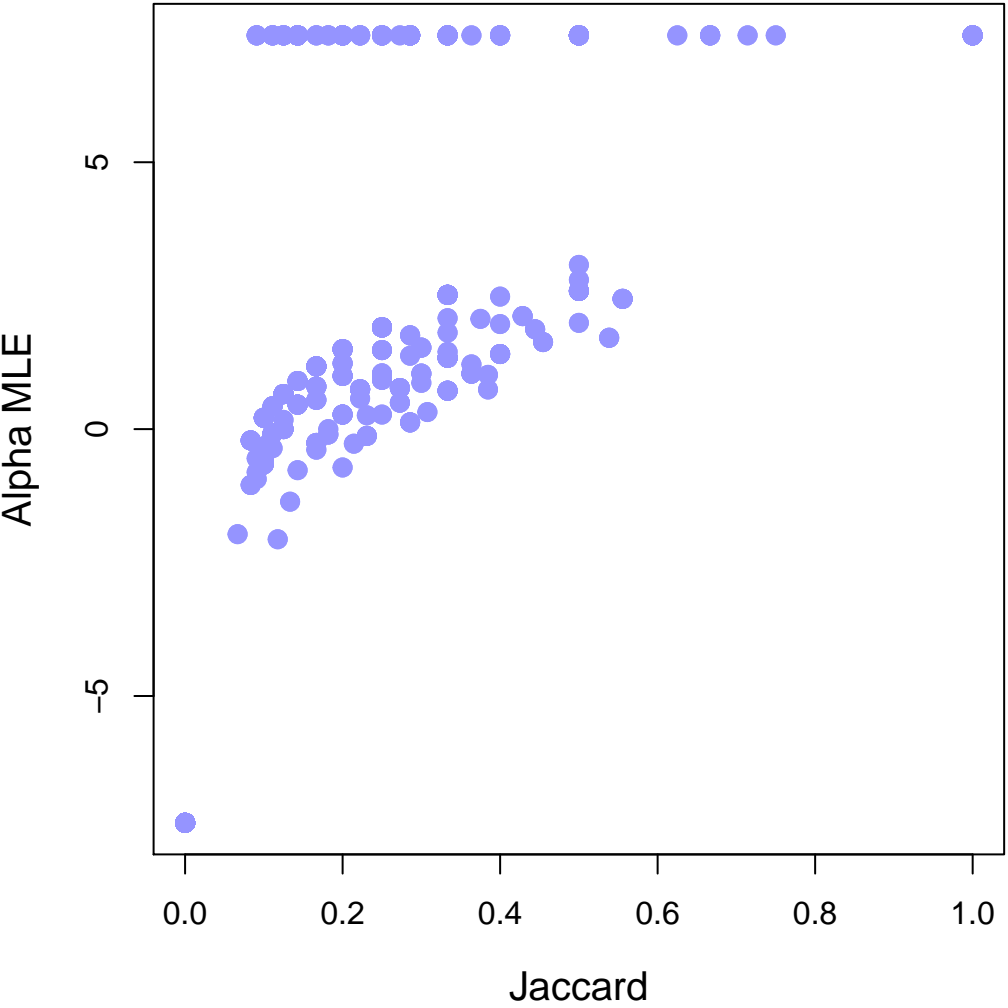

Supplement: Supplementary file 2 — Auxiliary Supplementary Materials and Other Supporting Files [file sciadv.abj9204_auxiliary_supplementary_materials_and_other_supporting_files.zip › codesupplement/plots/Fig S5 - jaccard by affinity.pdf]

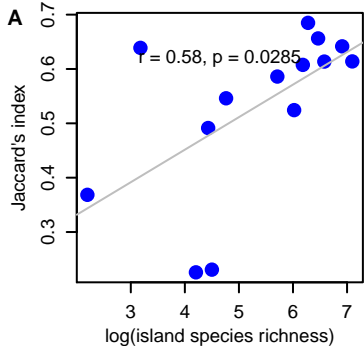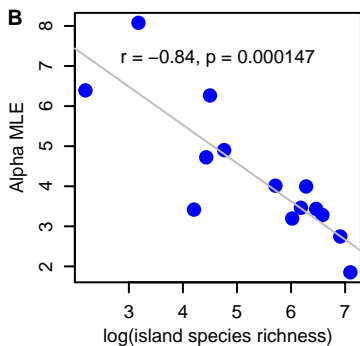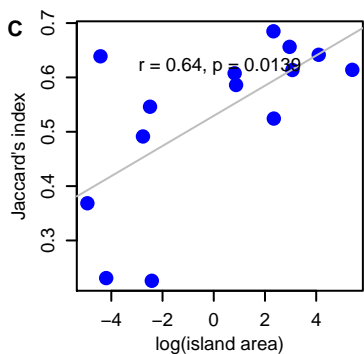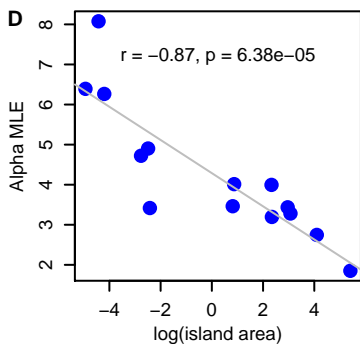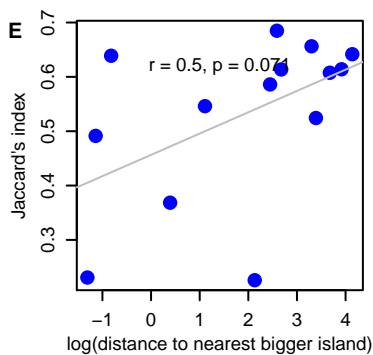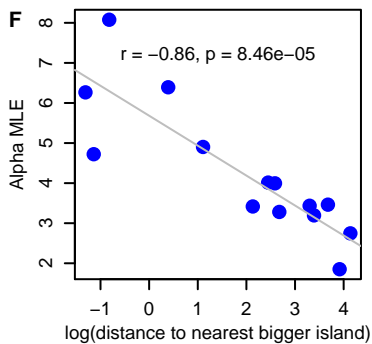

Supplement: Supplementary file 2 — Auxiliary Supplementary Materials and Other Supporting Files [file sciadv.abj9204_auxiliary_supplementary_materials_and_other_supporting_files.zip › codesupplement/plots/Fig 3 - Jaccard vs affinity for island area, distance and richness.pdf]

## mode of action of antibiotics

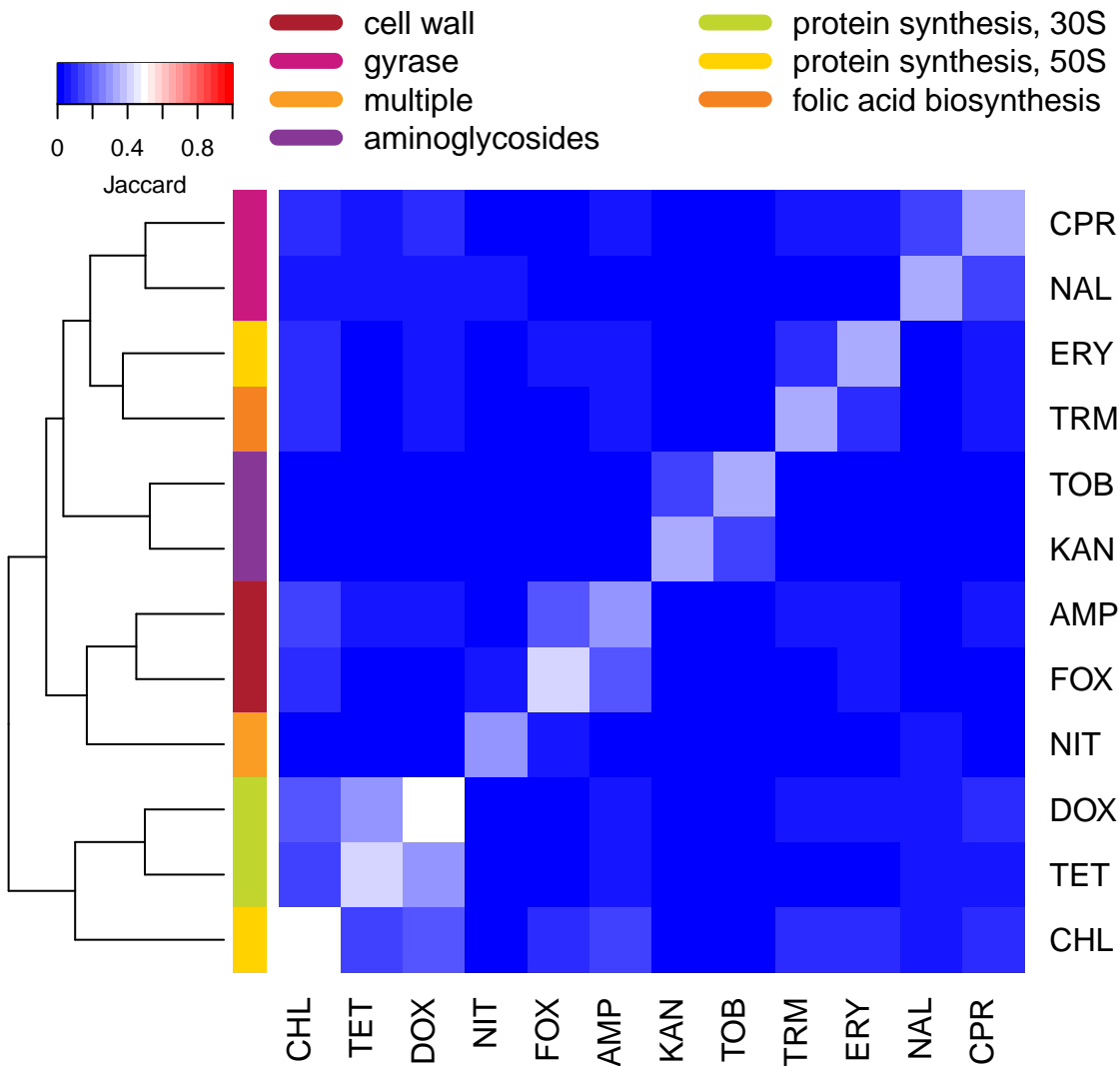

Supplement: Supplementary file 2 — Auxiliary Supplementary Materials and Other Supporting Files [file sciadv.abj9204_auxiliary_supplementary_materials_and_other_supporting_files.zip › codesupplement/plots/Fig S4A - antibiotic_Jaccard_completeplot_colorScaled.pdf]

**D**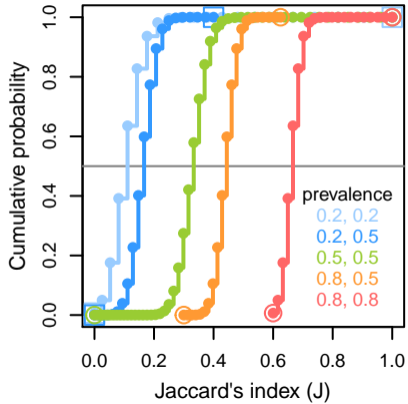**E**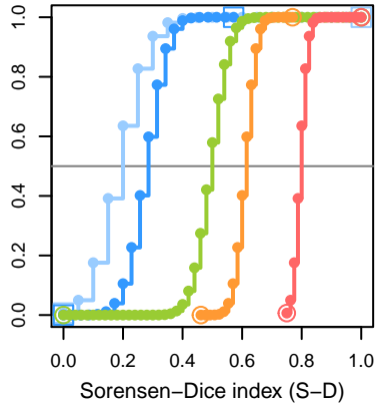**F**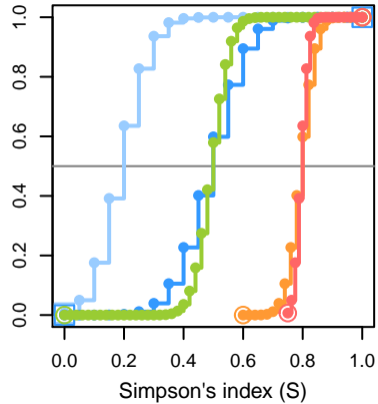

Supplement: Supplementary file 2 — Auxiliary Supplementary Materials and Other Supporting Files [file sciadv.abj9204_auxiliary_supplementary_materials_and_other_supporting_files.zip › codesupplement/plots/Fig 1 - cumulative prob of jaccard, sorensen and simpson.pdf]

**A**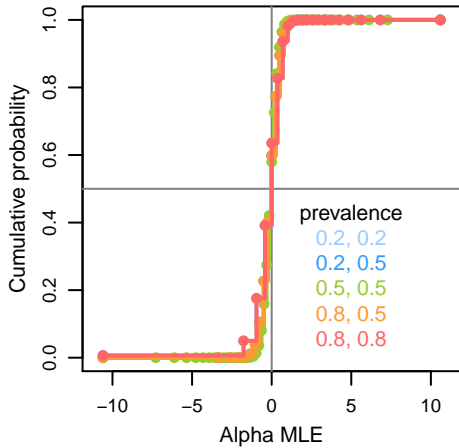**B**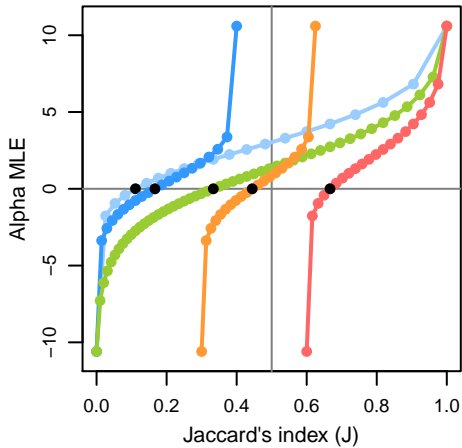

Supplement: Supplementary file 2 — Auxiliary Supplementary Materials and Other Supporting Files [file sciadv.abj9204_auxiliary_supplementary_materials_and_other_supporting_files.zip › codesupplement/plots/Fig 2 - cumulative prob of alpha_mle and some specific scanarios.pdf]

## Alpha MLE

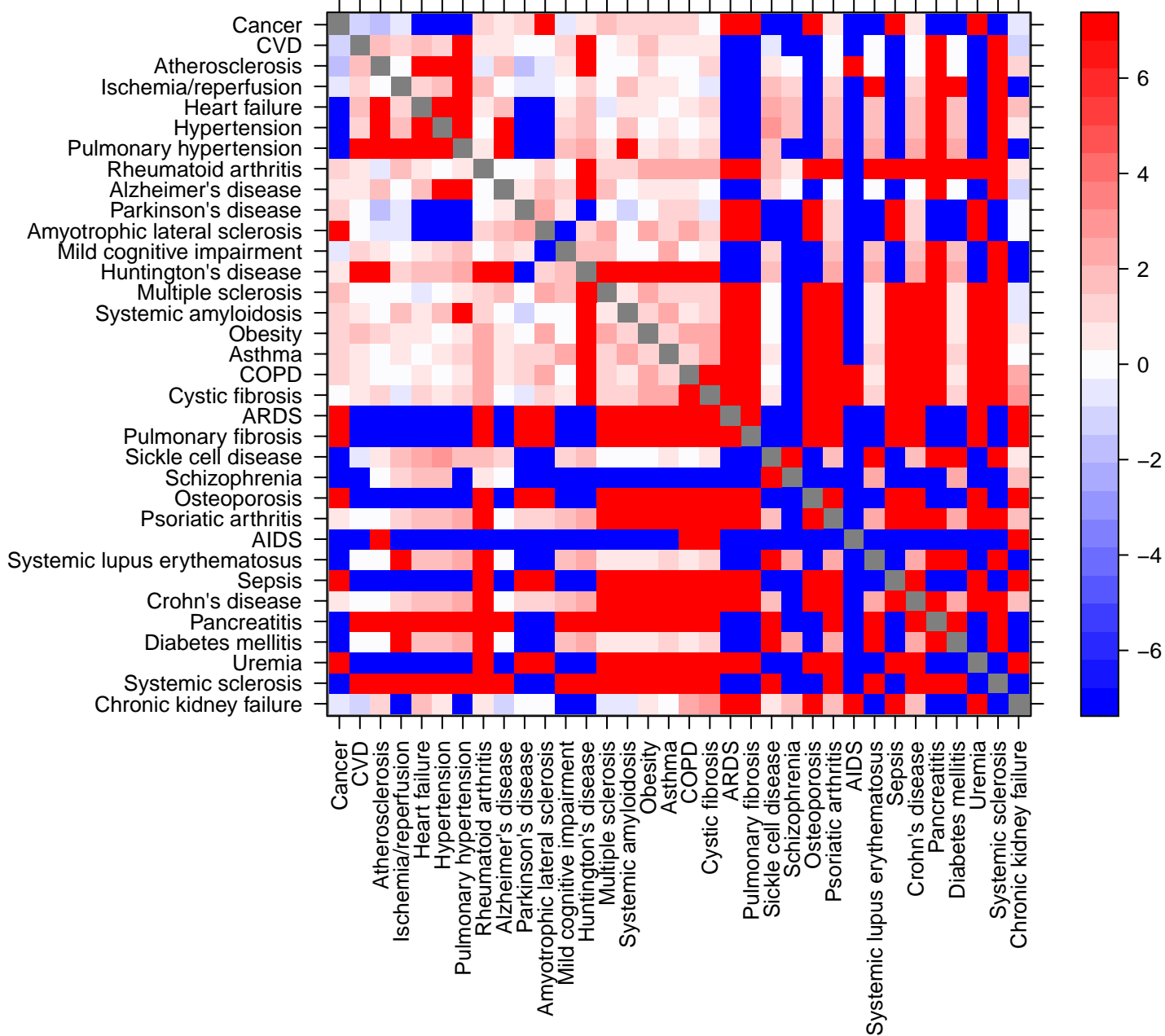

Supplement: Supplementary file 2 — Auxiliary Supplementary Materials and Other Supporting Files [file sciadv.abj9204_auxiliary_supplementary_materials_and_other_supporting_files.zip › codesupplement/plots/Fig S5 - AffinityMLE_levelplot.pdf]

# Alpha MLE

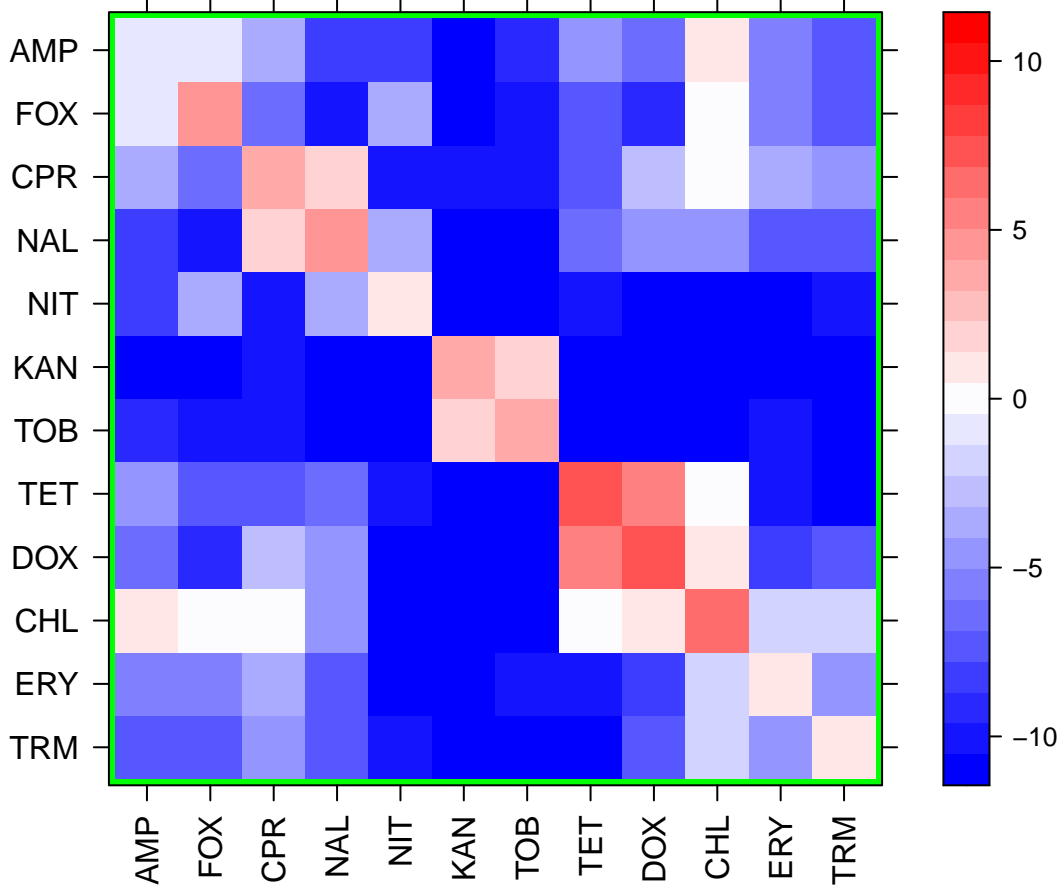

Supplement: Supplementary file 2 — Auxiliary Supplementary Materials and Other Supporting Files [file sciadv.abj9204_auxiliary_supplementary_materials_and_other_supporting_files.zip › codesupplement/plots/Fig S3D - antibiotic_AffinityMLE_newcolor_levelplot.pdf]

period 1830–1950 1951–2015

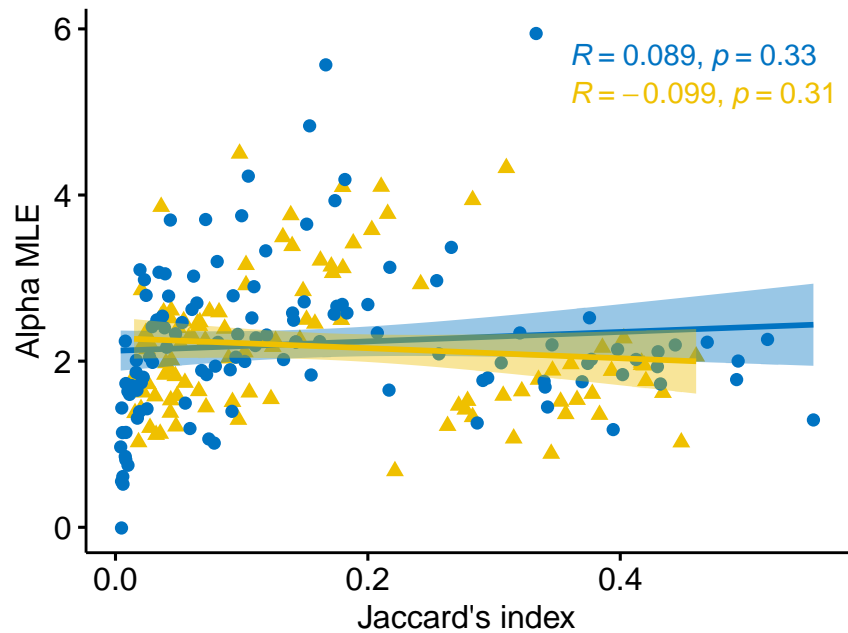

Supplement: Supplementary file 2 — Auxiliary Supplementary Materials and Other Supporting Files [file sciadv.abj9204_auxiliary_supplementary_materials_and_other_supporting_files.zip › codesupplement/plots/Fig 4A - spatial affinity - jaccard vs affinity.pdf]

# mode of action of antibiotics

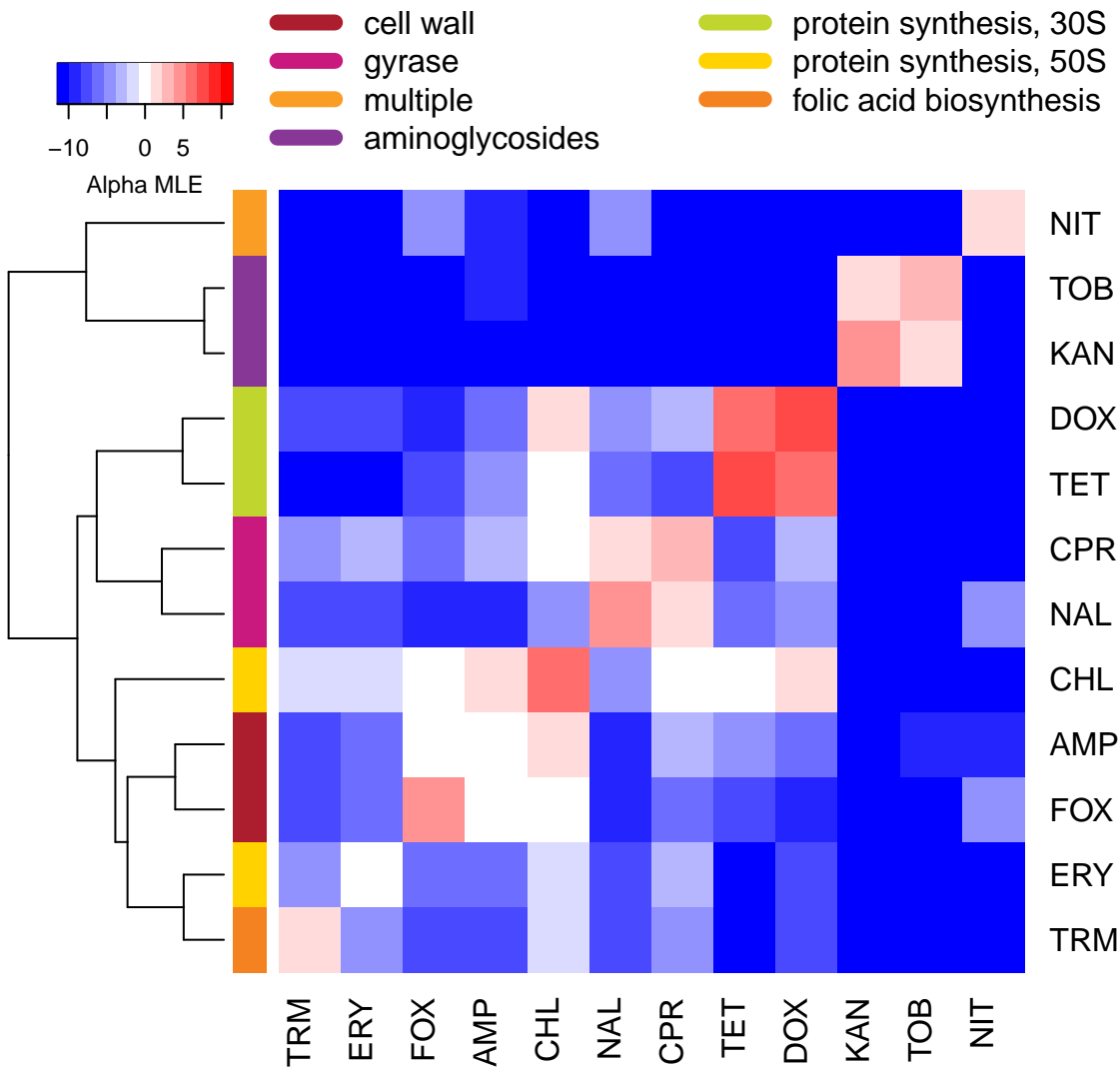

Supplement: Supplementary file 2 — Auxiliary Supplementary Materials and Other Supporting Files [file sciadv.abj9204_auxiliary_supplementary_materials_and_other_supporting_files.zip › codesupplement/plots/Fig S4B - antibiotic_AffinityMLE_completeplot.pdf]

# Jaccard

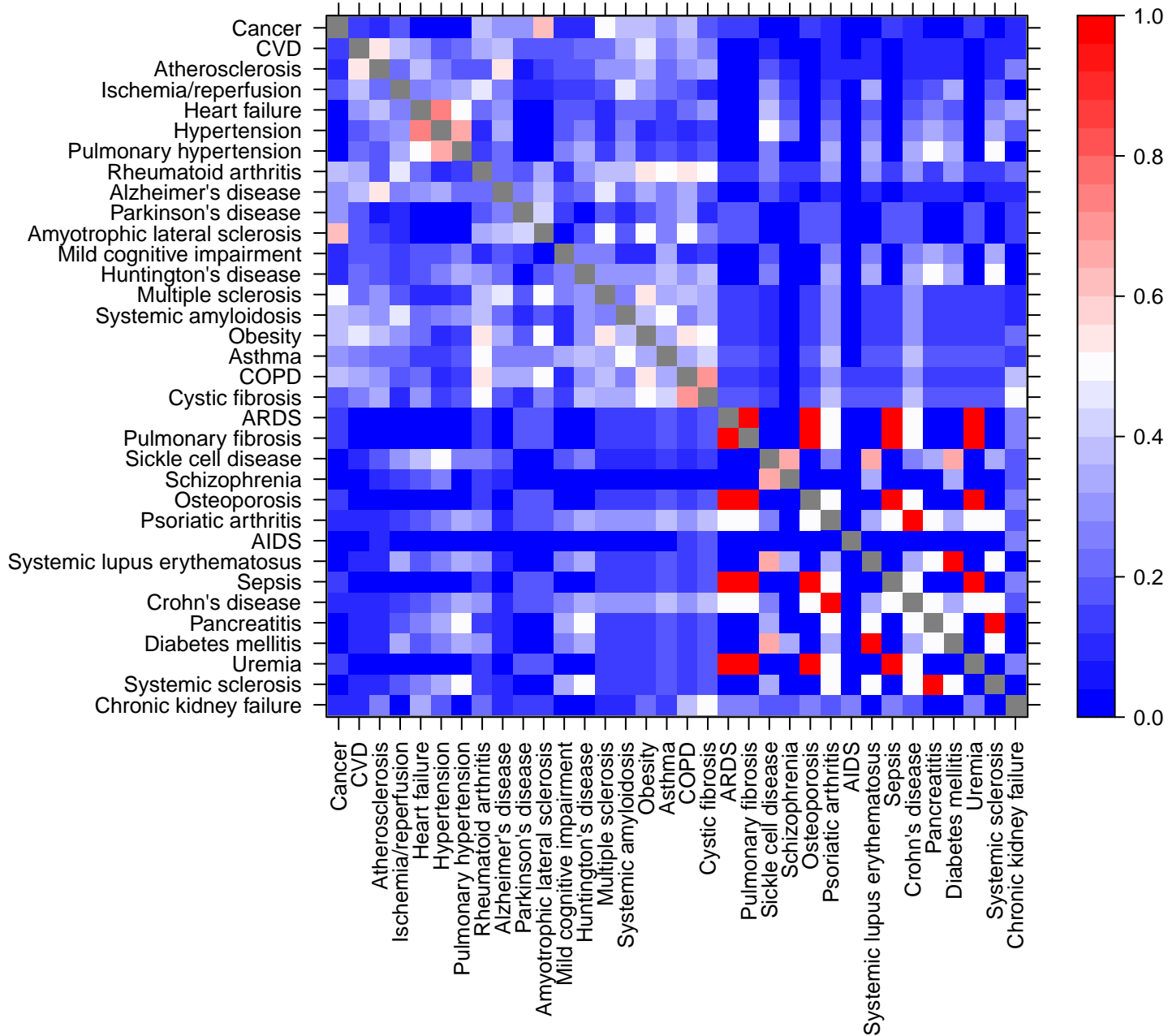

Supplement: Supplementary file 2 — Auxiliary Supplementary Materials and Other Supporting Files [file sciadv.abj9204_auxiliary_supplementary_materials_and_other_supporting_files.zip › codesupplement/plots/Fig S5 - Jaccard_levelplot.pdf]

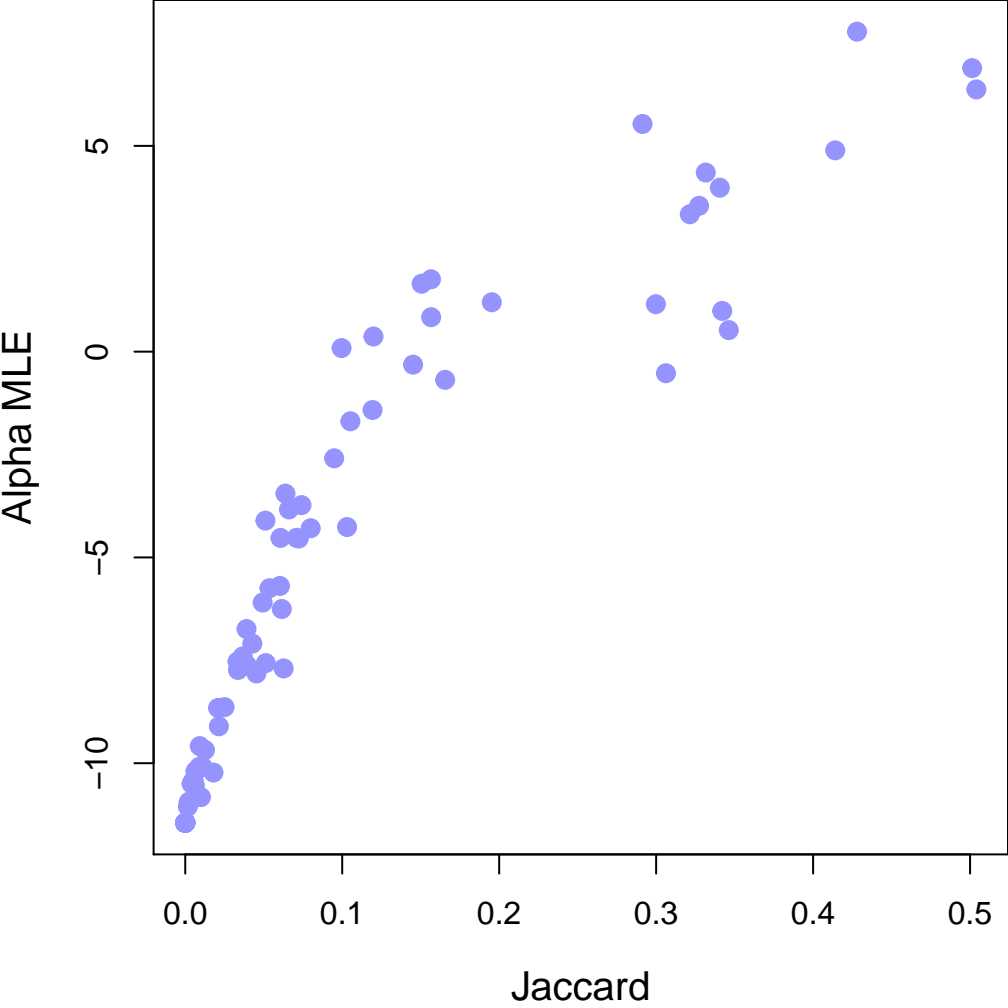

Supplement: Supplementary file 2 — Auxiliary Supplementary Materials and Other Supporting Files [file sciadv.abj9204_auxiliary_supplementary_materials_and_other_supporting_files.zip › codesupplement/plots/Fig S3A - antibiotic_Jaccard_vs_affinity.pdf]
